# Reconstructing Air Pollution Trends in Remote Forests of Central Europe Using Lichen Herbarium Specimens

**DOI:** 10.1007/s00244-025-01134-9

**Published:** 2025-06-21

**Authors:** Luca Paoli, Zuzana Fačkovcová, Anna Guttová

**Affiliations:** 1 Department of Biology, University of Pisa, Via L. Ghini 13, 56126 Pisa, Italy; 2 Plant Science and Biodiversity Centre, Slovak Academy of Sciences, Dúbravská Cesta 9, 84523 Bratislava, Slovakia

## Abstract

Lichen collections may contribute to a better understanding of past environmental conditions. By analysing herbarium specimens and recently collected material of the lichen *Lobaria pulmonaria*, this study reconstructs six decades (1960–2022) of air quality changes in remote forests of Central Europe (the Western Carpathians). Twenty specimens were selected for retrospective analysis of major and trace elements (Al, As, Cd, Cr, Cu, Fe, Hg, Mn, Ni, Pb, S, Sb, and Zn), focusing on both historical and current background concentrations. The results revealed peaks of potentially toxic elements (As, Fe, Hg, Mn, Ni, S, and Sb) up to 1989, with a progressive decline observed thereafter, particularly for Pb following the introduction of unleaded gasoline. Background element concentrations from 1960 to 1989 were at least four times higher than actual levels. The study highlights the value of herbarium collections for reconstructing air pollution history and tracking changes in background element concentrations in remote areas, particularly when alternative data sources are unavailable. It also addresses both the opportunities and limitations of this approach.

**Supplementary Information:**

The online version contains supplementary material available at 10.1007/s00244-025-01134-9.

Herbarium specimens provide valuable insights into reconstructing past environmental conditions, including heavy metal pollution (e.g. Rodríguez Martín et al. 2015; Rudin et al. 2017). Their significance in studying environmental changes related to heavy metal contamination has been demonstrated by several studies utilising plant (e.g. Herpin et al. 1997; Peñuelas and Filella 2002; Rodríguez Martín et al. 2015; Rudin et al. 2017), bryophyte (e.g. Weiss et al. 1999; Farmer et al. 2002; Peñuelas and Filella 2002; Shotbolt et al. 2007; Cao et al. 2008; Saxena et al. 2008; Foan et al. 2010; Ochota and Stebel 2013), and lichen specimens (e.g. Zschau et al. 2003; Purvis et al. 2007; Agnan et al. 2013; Minganti et al. 2014; Bajpai et al. 2016) for monitoring purposes. Herbarium specimens have been used to monitor different types of land use and environmental changes related to environmental pollution over time. Rudin et al. (2017) documented the decline of industrial pollution in Rhode Island (US) by measuring copper (Cu), lead (Pb), and zinc (Zn) in historical (1846–1916) and recent (2015) plant specimens. In India, Saxena et al. (2008) analysed moss collections to estimate the increase in metal pollution, particularly cadmium (Cd), iron (Fe), nickel (Ni), Pb, and Zn since 1895 in an urban agglomeration. Similarly, Cao et al. (2008) assessed spatial and temporal changes in heavy metal pollution in Shanghai (China) since 1965. Rodríguez Martín et al. (2015) determined concentrations of Cd, chromium (Cr), Ni, and Pb in herbarium specimens (leaves) collected in Valencia (Spain) in 1941 and again in 2012. Their findings showed an increase in most metals, reflecting urbanisation, while Pb levels decreased. In Poland, Ochota and Stebel (2013) reported a decline in Cd and Pb pollution between 1989 and 2012 using moss collections. Applications in rural areas also highlight significant trends. In Spain, Peñuelas and Filella (2002) observed increasing concentrations of heavy metals—aluminium (Al), barium Ba, Cd, Cr, Fe, Pb, strontium (Sr), and titanium (Ti) in vascular plants and bryophytes until the 1960s, followed by a gradual decline (1985–1995) with improved environmental quality. In the UK, Shotbolt et al. (2007) used herbarium moss specimens to reconstruct temporal trends (1851–2000) in heavy metal concentrations, identifying peaks in Cu and Pb during the late nineteenth century (coinciding with ore production peaks) and a subsequent decline. In Spain, Foan et al. (2010) used historical moss collections to investigate atmospheric deposition of polycyclic aromatic hydrocarbons (PAHs), highlighting an increasing trend in light PAHs. Further studies include Herpin et al. (1997), who reported fluctuations in heavy metals in mosses collected between 1845 and 1974 in Germany; Weiss et al. (1999) and Farmer et al. (2002) focused on Pb isotopes in *Sphagnum* specimens, providing valuable insights into pollution history.

Lichen and bryophyte collections are particularly valuable for reconstructing past atmospheric conditions, as these organisms rely on the atmosphere for their metabolism, providing a reliable indication environmental quality. Lichens, being perennial and slow-growing, do not shed thallus parts as readily as vascular plants. Moreover, their lack of a waxy cuticle and stomata facilitates the absorption of contaminants across the entire thallus surface (Hale 1983). As a result, lichens can accumulate elements to levels far exceeding their physiological needs, enabling the tracking of trace element deposition patterns over both spatial and temporal scales (Bačkor and Loppi 2009). Our study focuses on the epiphytic lichen *Lobaria pulmonaria* Hoffm., recognised for its sensitivity to air pollution, particularly SO₂ and heavy metals, as highlighted in lichen monitoring studies (e.g. Hawksworth and Rose 1970; Rose 1988; Ravera et al. 2023). The scientific literature has also documented the connection between deteriorating air quality and the retreat of this species in regions such as the Western Carpathians. A recent review by Paoli et al. (2021) summarised research by lichenologist Ivan Pišút and colleagues on the decline of air pollution-sensitive lichens in Central Europe (in terms of diversity and species frequency), with a focus on *L. pulmonaria*. During the twentieth century, the species experienced a significant decline in Slovakia, marked by substantial habitat loss and a contraction of its distributional range. Historically, its altitudinal range extended from 150 to 1400 m but is now primarily restricted to isolated, higher mountainous areas, with its lower altitudinal boundary at approximately 600 m. This decline has been attributed to a combination of factors, including air pollution, intensive agriculture, forest management practices (e.g. shortened logging periods and the conversion of beech forests to spruce), and habitat fragmentation (Liška and Pišút 1989). Today, *L. pulmonaria* occurs mainly in remote areas with low levels of environmental pollution and is regarded as a species closely associated with forests of high environmental quality and long-term ecological stability in the Western Carpathians (Paoli et al. 2021; Ravera et al. 2023). Given these considerations, analysing potentially toxic elements in herbarium specimens can provide valuable evidence of atmospheric contamination in remote areas. These areas, which lack direct pollution sources, likely reflect the impact of long-range transport of air pollutants. In this study, we examined *L. pulmonaria* collections from remote forests of the Western Carpathians and assessed newly collected material to characterise the past and present elemental profiles of the species in remote mountainous regions. The study aims to: 1) detect changes in air quality between 1960 and 2022, and 2) establish past and present background concentrations of selected heavy metals and metalloids in lichens of the Western Carpathians.

## Materials and Methods

### Experimental Constraints and Study Sites

*Lobaria pulmonaria* is a tripartite foliose lichen, with the green alga *Symbiochloris reticulata* (Tschermak-Woess) Skaloud, Friedl, A.Beck & Dal Grande as main photobiont and N-fixing cyanobacteria of the genus *Nostoc* as a secondary photobiont, confined to thallus structures called cephalodia. The mature thallus may exceed 20 or even 30 cm in diameter. Lobes are strap-shaped, 1–3 (up to 5) cm wide, mostly free from the substrate in their apical parts (Ravera et al. 2023).

**Fig. 1:**
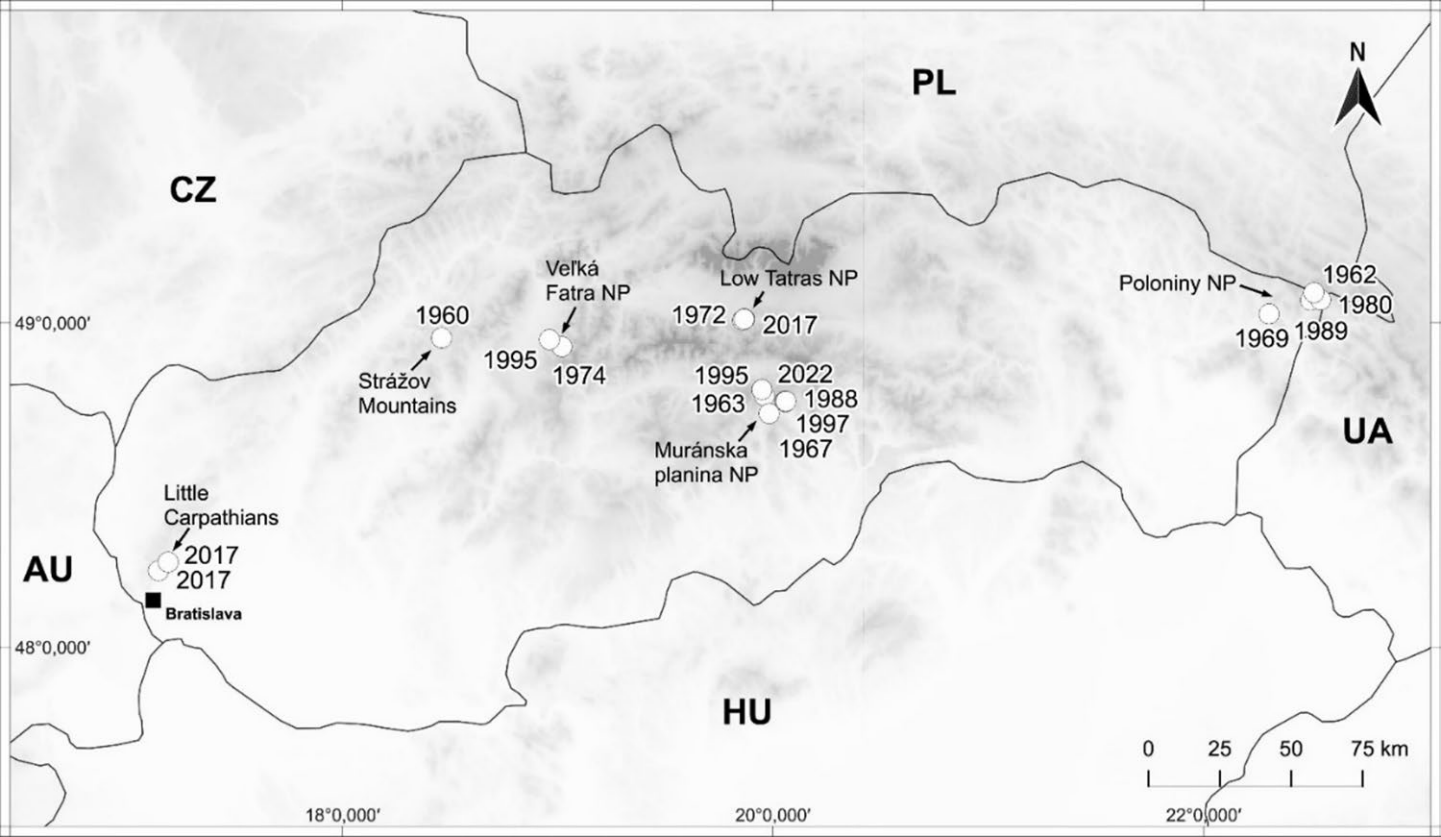
Sites of origin of *Lobaria pulmonaria* specimens in the Western Carpathians (Slovakia)

Fifteen *L. pulmonaria* specimens were carefully selected among those available in the collections of the Plant Science and Biodiversity Centre, Slovak Academy of Science (SAV) and the Natural History Museum, Bratislava (BRA). Specific details concerning selected herbarium specimens are reported in Supplementary Information (S1). Five further specimens refer to recently collected material (2017–2022). The collections of 2017 were part of a long-term translocation experiment (Paoli et al. 2020) and were included to provide an adequate spatial and temporal representation of air pollution in the Western Carpathians. Overall, all specimens, both older and recent collections, originate from remote forest sites in the mountains (mostly beech woods in natural parks) covering the whole range of the Western Carpathians in Slovakia, from the Little Carpathians in the west to the Poloniny Mts in the east (Fig. 1). All selected specimens were stored in paper envelopes. For each specimen, our aim was to prepare one analytical replicate (sample) without causing significant damage to the lichen specimens. As the procedure is destructive, only large specimens were included in the dataset. During the selection process, a small portion of *L. pulmonaria* lobes (up to 2 cm from the margin) was carefully harvested. The oldest collections (two specimens from the 1920s) were found to be contaminated with Hg, likely due to conservation treatments, and were excluded from further analysis. Based on the age of the remaining specimens, the following clusters (years) were established: 1960–1974, 1980–1989, 1995–1997, and 2017–2022. Each cluster is represented by at least three independent remote forests.

### Potentially Toxic Elements (PTEs)

To prepare each sample, generally about 100 mg of lichen material was carefully removed using plastic tweezers. Air-dried samples were cleaned manually (not washed) from extraneous material (e.g. bark pieces, soil particles, and moss fragments) and milled under liquid nitrogen using ceramic mortar and pestle, then mineralised with a mixture of 3 mL of 70% nitric acid (HNO_3_), 0.2 mL of 60% hydrogen fluoride (HF), and 0.5 mL of 30% hydrogen peroxide (H_2_O_2_) in a microwave digestion system (Milestone Ethos 900) at 280 °C and 55 bar. For each sample, thirteen elements of toxicological concern (namely, Al, arsenic (As), Cd, Cr, Cu, Fe, mercury (Hg), manganese (Mn), Ni, Pb, sulphur (S), antimony (Sb), and Zn) were quantified by inductively coupled plasma mass spectrometry (ICP-MS) using a PerkinElmer NexION 350 spectrometer (Waltham, MA, USA), and the concentrations were expressed on dry weight basis. The analytical quality was checked with the Standard Reference Material IAEA-336 (lichen) for Al, As, Cd, Cr, Cu, Fe, Mn, Pb, Sb, Zn, or alternatively GBW-07604 (poplar leaves) for Hg, Ni, and S (recoveries in the range 87–111%). One procedural blank and one sample of each certified material were included in each set of analysis (see Supplementary Materials S2 for details on Limits of Quantification and Standard Reference Materials). The precision of analysis (range of variation within 12%) was estimated by the variation coefficient of three replicates using fresh samples of *L. pulmonaria* from a remote forest site in Central Italy.

### Data Interpretation and Statistics

A bioaccumulation ratio (B ratio) was determined according to the suggestions reported in Cecconi et al. (2019), adapted to the constraints of our study. Such a parameter is a dimensionless ratio between species-specific element concentrations measured in native samples and the corresponding background values (Cecconi et al. 2019), used as a reference for proximate-natural, unaltered conditions. For practical reasons, the data were clustered according to the year of collection. Each cluster (1960–1974, 1980–1989, 1995–1997, 2017–2022) highlights a relevant period from the point of view of the known air pollution history in Central Europe (Paoli et al. 2021). Hence, in our study, to investigate the intensity of past air pollution, the first term of the B ratio represents the average concentrations of PTEs found in *L. pulmonaria* within each collection period (from 1960 to 1997), while the second term represents actual reference concentrations. Due to the limited number of suitable samples, the reference (background) values in *L. pulmonaria* were estimated using concentrations from recent collections in remote forests (2017–2022, see Table 1). The ratio can be interpreted in terms of bioaccumulation levels, based on the following intervals: Absence of bioaccumulation (≤ 1.0); Low bioaccumulation (> 1.0–2.1); Moderate (> 2.1–3.4); High (> 3.4–4.9); Severe (> 4.9).

**Table 1:** Concentrations (µg/g dw) of the investigated elements in *Lobaria pulmonaria*: site of origin (*NP* national park), altitude (approx. in m a.s.l.), and sampling year (further details in Supplementary Information S1)

Locality	Alt	Year	Al	As	Cd	Cr	Cu	Fe	Hg	Mn	Ni	Pb	S	Sb	Zn
1. Strážov Mountains	1100	1960	3037	12.82	0.57	3.49	19.2	2005	0.182	38.6	12.40	28.27	2852	1.52	105.9
2. Poloniny NP	1190	1962	880	4.02	0.25	0.92	13.9	630	0.206	102.5	1.75	5.89	2410	0.51	58.5
3. Muránska planina NP	930	1963	951	4.36	0.42	1.42	15.8	854	0.199	23.3	4.07	12.09	3279	1.54	73.1
4. Muránska planina NP	1000	1967	680	3.68	0.30	1.74	16.1	1036	0.140	27.5	8.54	10.22	3824	1.54	105.6
5. Poloniny NP	950	1969	936	4.82	0.67	1.40	25.1	454	0.021	43.4	3.64	9.57	2249	0.40	91.4
6. Low Tatras NP	800	1972	1693	2.16	0.21	2.17	12.4	2901	0.084	39.0	13.17	15.32	2088	1.34	100.5
7. Veľká Fatra NP	850	1974	1406	8.11	0.27	1.82	11.5	1301	0.215	24.2	6.26	11.42	2632	2.74	75.6
8. Poloniny NP	800	1980	636	4.16	0.41	1.29	12.8	789	0.130	26.5	2.33	9.28	2472	1.26	92.0
9. Muránska planina NP	900	1988	1385	3.38	0.49	1.34	18.6	528	0.106	19.0	8.03	9.35	2618	0.73	69.7
10. Poloniny NP	1070	1989	1319	4.15	1.21	1.87	15.3	1065	0.088	735.9	2.54	58.42	1917	0.77	120.7
11. Veľká Fatra NP	650	1995	2198	3.03	0.61	1.39	23.4	635	0.109	21.8	3.71	5.07	2386	0.63	145.3
12. Muránska planina NP	800	1995	976	2.06	0.27	1.04	14.0	479	0.072	17.1	4.14	6.30	1425	0.44	69.8
13. Muránska planina NP	920	1997	1222	5.89	0.33	1.80	32.9	1053	0.118	26.3	4.10	11.12	2014	0.76	170.4
14. Low Tatras NP	720	2017	404	0.07	0.86	2.26	7.7	295	0.038	34.6	2.03	1.45	1273	0.14	114.9
15. Low Tatras NP	750	2017	522	0.18	0.58	2.28	7.9	384	0.038	44.9	3.38	1.69	1293	0.14	105.1
16. Little Carpathians	450	2017	557	0.3	0.47	1.79	7.5	410	0.056	82.1	1.39	4.19	1327	0.27	62.9
17. Little Carpathians	300	2017	551	0.21	0.45	1.55	8.0	405	0.058	46.6	1.78	3.13	1460	0.31	74.8
18. Muránska planina NP	900	2022	393	0.54	0.23	0.50	7.5	297	0.040	16.4	1.04	1.42	1492	0.16	36.8

A pollution load index (PLI) was calculated for the collections 1960–1974, 1980–1989, and 1995–1997 according to the formula PLI = (PI_1_ × PI_2_ × PI_3_ × … × PI_n_)^1/n^, where n (13) is the number of potentially toxic elements considered, and PI is represented here by the B ratio of each element (determined with reference to recently collected specimens, 2017–2022). Element deposition patterns were analysed using hierarchical clustering, with Pearson’s correlation coefficients as the similarity metric and average linkage as the agglomeration method. Non-parametric statistics (Mann–Whitney U test,* p* < 0.05) were used to check for each element significant differences between collection periods. Statistical analyses were conducted with the software STATISTICA 7.0.

## Results

**Fig. 2:**
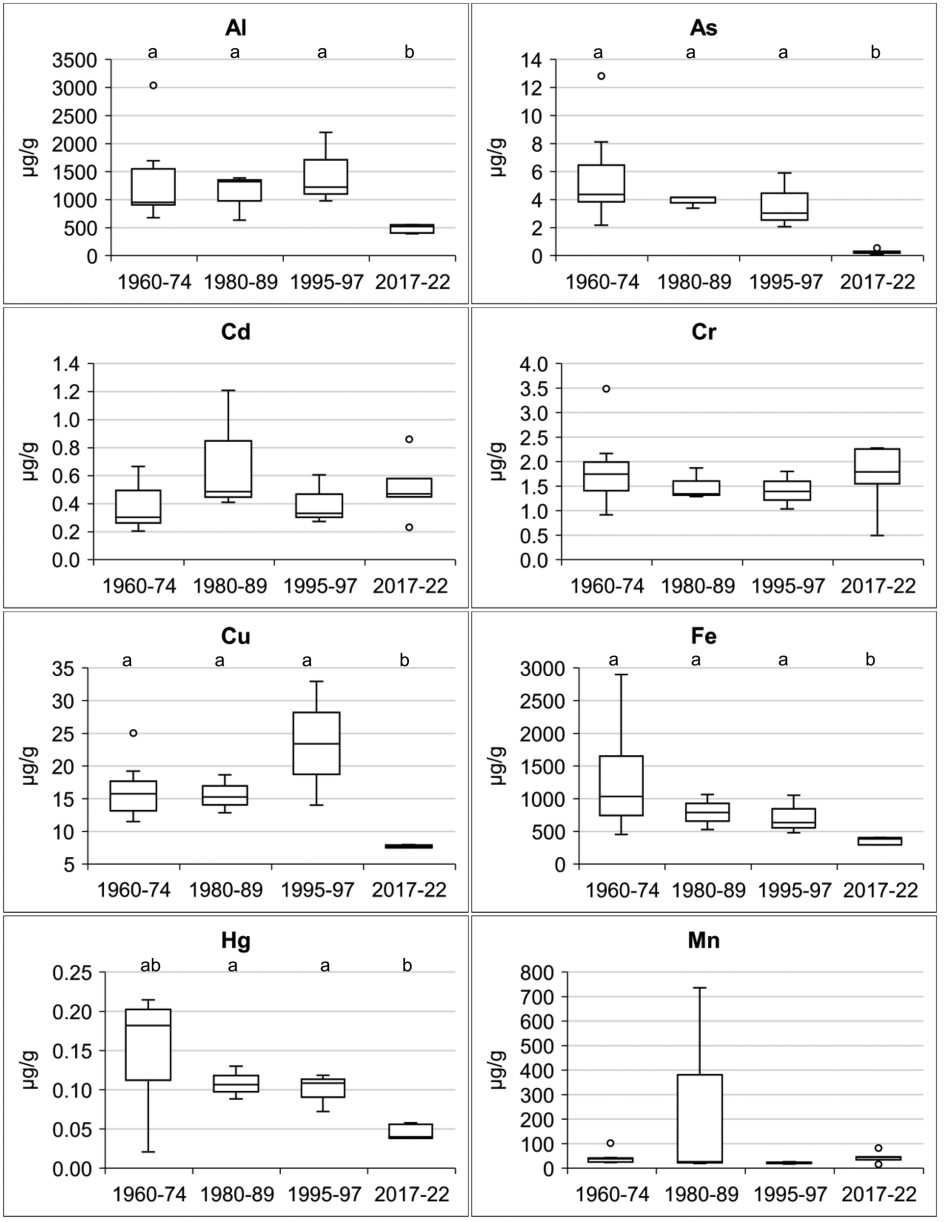

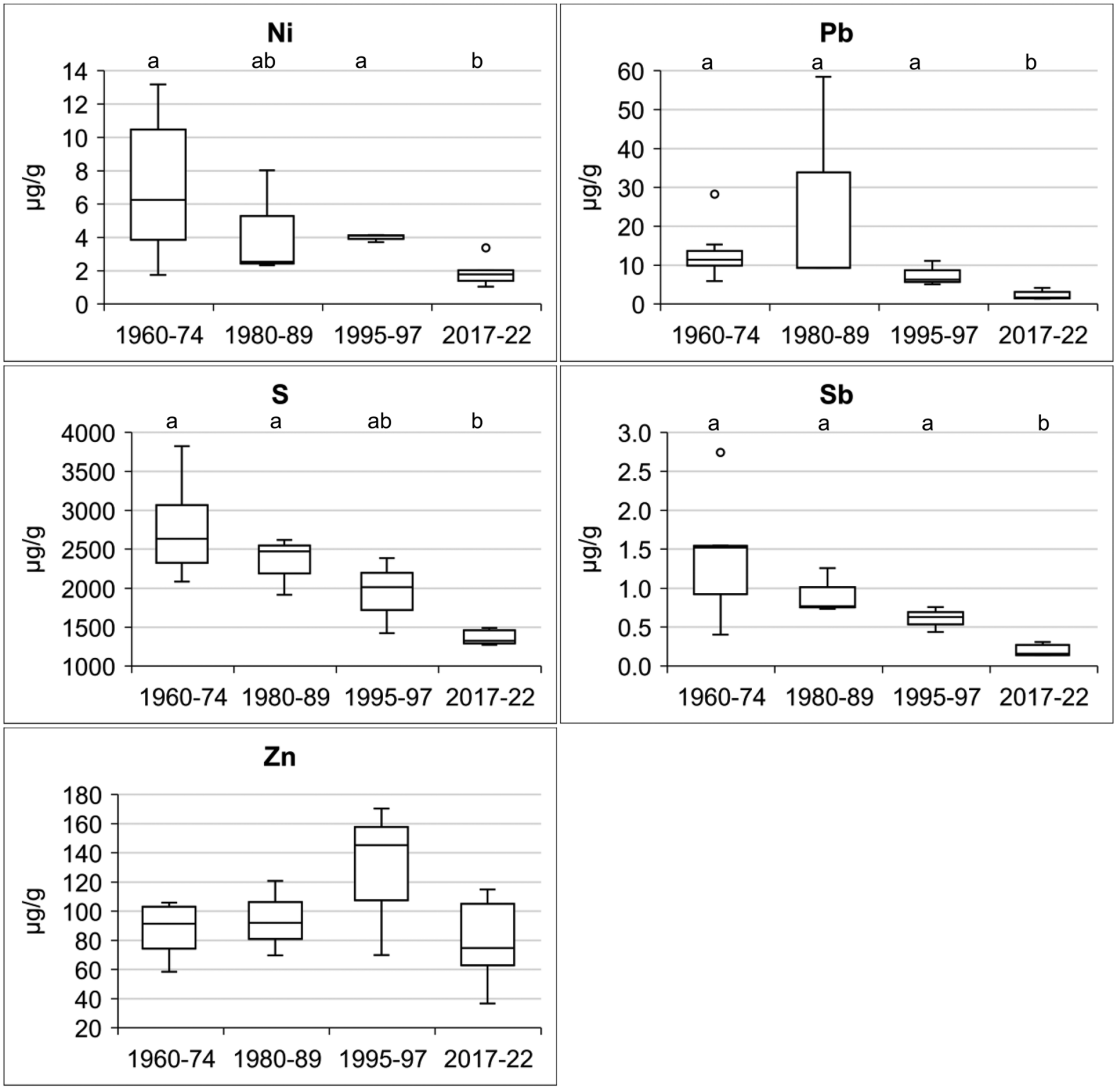
Box plots of the concentrations of major and trace elements between the study periods. Box: median value, 25th–75th percentiles. Whiskers: minimum and maximum values within 1.5 times the interquartile range. Eventual outliers are depicted as individual points beyond the whiskers. Groups labelled by a different letter are significantly different (*p* < 0.05)

The concentrations of Al, As, Cd, Cr, Cu, Fe, Hg, Mn, Ni, Pb, S, Sb, and Zn in *L. pulmonaria* are reported in Table 1, along with the site of origin and sampling year. Figure 2 summarises the concentrations of major and trace elements across the study periods. Peaks in As, Fe, Hg, Mn, Ni, Pb, S, and Sb concentrations were observed during 1960–1989, followed by a significant decrease in recent collections. A progressive decline was particularly evident for As, Fe, Hg, Ni, Pb, S, and Sb. A distinct reduction in Pb concentrations after 1989 (notably in collections from 1995–1997 onward) appears linked to the introduction of unleaded gasoline. The average pollution load index (PLI) reflects the overall trend in air pollution, with values of 2.7 in the 1960s–1970s, increasing slightly to 2.8 in the 1980s before declining to 2.0 in the 1990s and reaching its lowest values in recently collected specimens. These recent specimens are characterised by reduced concentrations of most elements, except for Cd, Cr, and occasionally Mn.

Table 2 shows the bioaccumulation (B) ratios for the period 1960–1997 (averaged per study site and time period), with recent collections (2017–2022) serving as a reference background (B ratio = 1). The greatest deviations in B ratios between past and present concentrations were observed for As (14.0–21.9), Pb (3.2–10.8), and Sb (3.0–6.7). According to the classification intervals established by Cecconi et al. (2019), some mountain forests experienced the highest levels of deposition across all investigated elements. These include the Strážov Mountains in Central Slovakia (average B ratio of 7.8, with a peak of 49.2 for As in 1960), the Veľká Fatra Mountains in Northern Slovakia (average B ratio of 5.4, with a peak of 31.1 for As in 1974), and the Poloniny Mountains in Eastern Slovakia (average B ratio of 6.0, with a peak of 24.6 for Pb in 1988). Extremely high levels of Hg (up to 10 µg/g dw) were recorded in two older specimens, but were excluded from the analysis, as they likely reflected treatment with mercuric chloride (HgCl_2_), a substance commonly used in the past as an insecticide to protect herbarium collections.

**Table 2:** Bioaccumulation (B) ratios in *Lobaria pulmonaria* specimens between 1960 and 1997 (averaged per study site and period), calculated with reference to recent collections (2017–2022)

Locality	Year	Al	As	Cd	Cr	Cu	Fe	Hg	Mn	Ni	Pb	S	Sb	Zn	avg
1. Strážov Mountains	1960	6.3	49.2	1.1	2.1	2.5	5.6	4.0	0.9	6.4	11.9	2.1	7.5	1.3	7.8
2. Poloniny NP	1962	1.8	15.4	0.5	0.5	1.8	1.8	4.5	2.3	0.9	2.5	1.8	2.5	0.7	2.8
3. Muránska planina NP	1963	2.0	16.7	0.8	0.8	2.0	2.4	4.3	0.5	2.1	5.1	2.4	7.6	0.9	3.7
4. Muránska planina NP	1967	1.4	14.1	0.6	1.0	2.1	2.9	3.0	0.6	4.4	4.3	2.8	7.6	1.3	3.6
5. Poloniny NP	1969	1.9	18.5	1.3	0.8	3.2	1.3	0.5	1.0	1.9	4.0	1.6	2.0	1.2	3.0
6. Low Tatras NP	1972	3.5	8.3	0.4	1.3	1.6	8.1	1.8	0.9	6.8	6.4	1.5	6.6	1.3	3.7
7. Veľká Fatra NP	1974	2.9	31.1	0.5	1.1	1.5	3.6	4.7	0.5	3.3	4.8	1.9	13.5	1.0	5.4
8. Poloniny NP	1980	1.3	16.0	0.8	0.8	1.7	2.2	2.8	0.6	1.2	3.9	1.8	6.2	1.2	3.1
9. Muránska planina NP	1988	2.9	13.0	0.9	0.8	2.4	1.5	2.3	0.4	4.2	3.9	1.9	3.6	0.9	3.0
10. Poloniny NP	1989	2.7	15.9	2.3	1.1	2.0	3.0	1.9	16.4	1.3	24.6	1.4	3.8	1.5	6.0
11. Veľká Fatra NP	1995	4.5	11.6	1.2	0.8	3.0	1.8	2.4	0.5	1.9	2.1	1.7	3.1	1.8	2.8
12. Muránska planina NP	1995	2.0	7.9	0.5	0.6	1.8	1.3	1.6	0.4	2.2	2.7	1.0	2.2	0.9	1.9
13. Muránska planina NP	1997	2.5	22.6	0.6	1.1	4.3	2.9	2.6	0.6	2.1	4.7	1.5	3.7	2.2	4.0
1960–1974	avg B	2.8	21.9	0.7	1.1	2.1	3.7	3.3	0.9	3.7	5.6	2.0	6.7	1.1	4.3
1980–1989	avg B	2.3	15.0	1.4	0.9	2.0	2.2	2.4	5.8	2.2	10.8	1.7	4.5	1.2	4.0
1995–1997	avg B	3.0	14.0	0.8	0.8	3.0	2.0	2.2	0.5	2.1	3.2	1.4	3.0	1.6	2.9

## Discussion

### Air Quality Trends in Remote Areas of the Western Carpathians

The Western Carpathians, including Slovakia, were subjected to elevated emissions of sulphur dioxide (SO_2_), nitrogen oxides (NO_x_), and heavy metals during the second half of the twentieth century. This significantly deteriorated air quality in both urban and remote areas until the 1980s. However, emission trends in Slovakia shifted significantly after the sociopolitical changes of 1989, leading to a general decline in emissions from the 1990s onwards (Mladý 2019). The historical prevalence of acidic pollution in the Western Carpathians also shaped epiphytic lichen communities in forests, which are dominated by acidophilus and oligotrophic crustose/foliose species, with a reduced presence of fruticose forms (Guttová et al. 2017). According to Svoboda et al. (2011), forest degradation and air pollution throughout the last century were the primary reasons for the scarcity (or even complete absence) of sensitive indicator species (such as *L. pulmonaria*) characteristic of high-quality environments in some areas of the Western Carpathians.

In fact, the rapid decline of indicator species (including *L. pulmonaria*) has been traditionally related to the rising levels of SO_2_ air pollution and the disturbance of the woodland microclimate (Hawksworth and Rose 1970; Rose 1976). Sulphur emissions have been generally associated with fossil fuels combustion, especially in the past. In our study, recent *L. pulmonaria* records (2017–2022) exhibited S levels in the range of 1300–1500 µg/g, while older records (1960–1997) were enriched, with concentrations between 1400 and 3800 µg/g, reflecting the overall decrease in S emissions in recent years. According to Richardson (1981), background S levels in lichens generally remain below 1000 µg/g, while thalli with concentrations exceeding 2000 µg/g are considered enriched (Nieboer et al. 1978). Sulphur content in lichens varies depending on the species. In Slovakia, Bačkor et al. (2003) measured 3900 µg/g S in the crustose lichen *Lecanora chlarotera* and 1400 µg/g in the foliose *Physcia tenella* in areas affected by S emissions from combustion processes. Similarly, Paoli et al. (2014) reported 2940–5720 µg/g S in the foliose lichen *Xanthoria parietina* in a rural area of the Little Carpathians impacted by S deposition near a cement mill, including remote sites. In the same area, transplants of the fruticose lichen *Evernia prunastri* exposed for up to six months showed S levels of 2070–2490 µg/g (Paoli et al. 2017). It is worth noting that while *L. pulmonaria* is highly sensitive to S and phytotoxic gases, making it a reliable bioindicator of their effects, it may not be an ideal bioaccumulator since it tends to disappear from polluted areas. Despite this limitation, *L. pulmonaria* has been extensively used as a monitoring tool in forests worldwide for assessing various air pollution impacts. These include polycyclic aromatic hydrocarbons (Blasco et al. 2011), heavy metals (Chahloul et al. 2022; Paoli et al. 2020), long-range radionuclide transport (Loppi and De Dominicis 1996; Riga-Karandinos and Karandinos 1998), acid rain deposition (Farmer et al. 1991, 1992), and pollution from local sources (e.g. Kouadria et al. 2021; Riga-Karandinos and Karandinos 1998; Yemets et al. 2014).

In our study, major and trace element concentrations in *L. pulmonaria* collections were used as proxies for heavy metal pollution in remote areas, likely reflecting long-range atmospheric transport. The overall trend of decreasing concentrations prompted the calculation of Bioaccumulation (B) ratios and the pollution load index (PLI), using recent collections as a reference. A clear trend of decreasing Pb concentrations was observed, after peaks around 1989 (collections from 1995 to 1997 and later) likely reflecting the introduction of unleaded gasoline. The positive impact of this policy was evident even in lichens from remote areas of Central Europe, such as the Western Carpathians, as indicated by the reduced Pb content in recent collections. Further evidence of the decline in heavy metal pollution was corroborated by lichen transplant studies, which demonstrated significant reductions in Cu, Cd, Cr, Mn, Ni, Pb, and Zn levels in urban areas of Slovakia compared to pre-1990 conditions (Guttová et al. 2011). Some of the peaks of elements observed in our *L. pulmonaria* specimens could be explained by the presence of industrial sources of air pollution located in nearby rural areas. One of such examples is from the Orava region (Northern Slovakia), an area extensively concerned in the past by air pollution from heavy metals, metalloids and SO_2_ from the metallurgical industry (Kontrišová and Lackovičová 1989). In particular, high values of As (up to 9.2 μg/g) were reported in the fruticose lichen *Hypogymnia physodes* (between 1985 and 1986) by Kontrišová and Lackovičová (1989), in line with the peak of As (8.1 μg/g) observed in 1974 in our *L. pulmonaria* in the nearby Veľká Fatra Mountains.

**Fig. 3:**
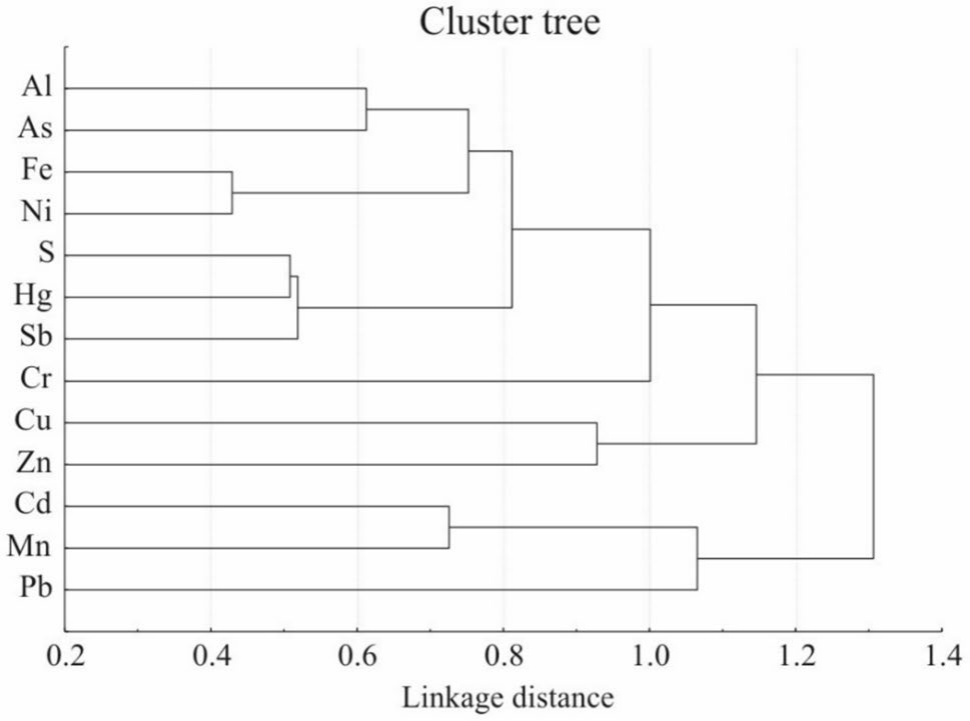
Cluster tree of element depositions performed by the average linkage method on the basis of Pearson’s correlation coefficient

A cluster analysis was conducted on the concentration data to identify groups of elements with similar patterns (Fig. 3).

As noted by Minganti et al. (2014) in relation to lichens, comparing cluster trees across studies can be challenging due to differences in species, elements, natural and anthropogenic characteristics of the studied sites, as well as the clustering techniques employed. Moreover, many elements may originate from multiple sources (Bargagli 1998). In our dataset, two closely related clusters comprising Al, As, Fe, and Ni likely reflect both the resuspension of soil particles and the possible influence of metal works in nearby areas. The cluster formed by S and Hg appears to be associated with combustion processes and long-range transport (Bargagli 1998). This cluster also seems to be closely linked with Sb, which can be naturally released from bedrock and volcanic activity, including geothermal sources (Adriano 1986). Anthropogenic sources of Sb in the atmosphere may include vehicle component wear (Fujiwara et al. 2011) and fuel combustion (Sternbeck et al. 2002). Another cluster, represented by Cu and Zn, includes essential micronutrients for plants and lichens that are potentially harmful at elevated concentrations. These elements may originate from motor vehicles and metal works (Bargagli 1998). The cluster comprising Cd, Mn, and Pb may reflect multiple sources, including soil resuspension (Mn and Pb) and motor vehicles (Cd and Pb). Indeed, despite the overall trend of decreasing pollution in Slovakia, significant peaks in element deposition have been reported recently, based on lichen monitoring in urban areas (Demková et al. 2019a) and around local pollution sources in rural settings (Demková et al. 2019b).

In our study, two additional specimens collected in the Little Carpathians (near Bratislava) by the naturalist Karol Mergl at the beginning of the last century (exact date not specified on the envelope) were found to be contaminated with Hg and were therefore excluded from the dataset. Elevated levels of Hg in older specimens often reflect the use of HgCl_2_, a chemical commonly applied in the past as an insecticide to protect herbarium collections from insect damage. All remaining specimens were confirmed to have been stored without prior chemical treatment with Hg.

### Background Element Concentrations in *L. pulmonaria*

Older lichen collections, combined with recent material, provide valuable insights into past background element concentrations and their changes over time in remote areas. Despite the limited dataset, most elements measured in our study (with the exceptions of Cd, Cr, and occasionally Mn) exhibited a clear decreasing trend over time, with the lowest values recorded in specimens collected between 2017 and 2022. Overall, the elemental content of *L. pulmonaria* from remote forests between 1960 and 1989 was at least four times higher than current levels, while values recorded up to 1997 were approximately three times higher. Locally, element concentrations in samples from remote areas may exceed background reference levels due to geochemical anomalies and/or the long-range transport of pollutants (Frati et al. 2005). For example, Mn concentrations in certain parts of the Carpathians, such as the Little Carpathians, are influenced by the natural geological characteristics of these sites (e.g. Paoli et al. 2014, 2020). Moreover, a wide range of industrial pollution sources, even in rural areas, likely impacted air quality and the major and trace element content of lichens, particularly in the past. These sources included mining activities (Demková et al. 2019b), fertiliser production facilities (Jelínková 1973), steel and metallurgical factories (Dzubaj et al. 2008; Kontrišová and Lackovičová 1989), copper and mercury smelters (Banásová et al. 2010; Lackovičová et al. 1994), cement and magnesite factories (Paoli et al. 2014; Pišút and Pišút 2006), and superphosphate (Kaleta 1973) and aluminium production facilities (Pišút and Lisická-Jelínková 1974). A comparison of our data with the concentrations of the same elements in *L. pulmonaria* from other remote areas of the Northern Hemisphere is provided in Table 3. For an appropriate temporal comparison, our data have been divided into past and recent collections. Recent records (2017–2022) align with concentrations reported in remote forests with low air pollution in the Mediterranean and boreal zones of Europe (Bolshunova et al. 2018; Paoli et al. 2020), except for Zn, which is generally higher in the Slovak samples. In contrast, for certain toxicologically significant elements (e.g. Fe, Pb, S), older Slovak samples (1960–1997) exhibit values comparable to those reported from areas affected by anthropogenic disturbances, such as Balkan localities (Riga-Karandinos and Karandinos 1998). In a recent study, Paoli et al. (2020) exposed *L. pulmonaria* in selected beech forests of the Western Carpathians to investigate whether current air quality continues to hinder recolonization in sites where the species disappeared over the past century. Their findings revealed significant accumulation of various PTEs (namely, As, Mn, Ni, Pb, S, Sb, Zn, and occasionally Cd, Cr, and Cu) in the thalli, along with a strong correlation between stress symptoms and current air quality. These results suggest that atmospheric pollution, in conjunction with microclimatic factors, may still limit the recolonization of *L. pulmonaria* in parts of the Western Carpathians.

**Table 3:** Concentrations of potentially toxic elements in *Lobaria pulmonaria* from remote areas in Europe and beyond: comparison with our data. For each study, sampling site, year, number of samples (n), range of values (min–max), and/or mean concentrations (µg/g dw) are reported

Reference	Site	Year(s)	n	Al	As	Cd	Cr	Cu	Fe	Hg	Mn	Ni	Pb	S	Sb	Zn
Riga-Karandinos & Karandinos (1998)	Greece	n.a.*	22	–	–	1.56–6.40 3.42	–	4.6–12.3 6.8	339–2180 1103	–	17.7–137.1 65.3	–	3.9–21.1 9.8	1700–4200 2700	–	16.9–59.4 28.2
Bolshunova et al. (2018)	Baikal Lake	2015	9	228–800 505	0.29–0.77 0.44	0.04–0.36 0.14	3.6–7.2 5.0	4.2–8.3 5.9	171–453 301	0.03–0.09 0.06	17–218 84	0.42–1.39 0.82	0.72–2.32	–	0.006–0.078 0.043	35.1–55.4 46.1
Paoli et al. (2020)	Italy	2017	3	536	0.13	0.66	1.7	6.0	–	–	53	1.4	1.3	1042	0.08	19
Chahloul et al. (2022)	Tunisia	2020	2	1151–1979	2.24	0.11–0.17	3.0–4.7	11.2	1151–1637	–	–	2.1–5.4	2.5–2.9	–	–	21.9–44.8
This study (past)	Slovakia	1960–1997	13	636–3037 1332	2.06–12.82 4.82	0.21–1.21 0.46	0.9–3.5 1.7	11.5–32.9 17.8	454–2091 1056	0.021–0.215 0.129	17.1–735.9 88.1	1.75–13.17 5.75	5.1–58.4 14.8	1425–3824 2474	0.40–2.74 1.09	58.5–170.4 98.4
This study (recent)	Slovakia	2017–2022	5	393–557 485	0.07–0.54 0.26	0.23–0.86 0.52	0.5–2.3 1.7	7.5–8.0 7.7	295–410 358	0.038–0.058 0.046	16.4–82.1 44.9	1.04–3.38 1.92	1.4–4.2 2.4	1273–1492 1369	0.14–0.31 0.20	36.8–114.9 78.9

^*^n.a. = not available

### Lichenological Studies and Constraints in Using Herbarium Collections to Reconstruct Air Pollution Trends

With regard to lichens from herbarium collections used as biomonitors of heavy metal deposition, Zschau et al. (2003) in the US examined specimens of the epilithic genus *Xanthoparmelia* (collected in 1975–1976) from six localities to explore temporal trends of heavy metals, comparing them with samples collected in 1998. They reported site-dependent variations but observed a general decrease in Pb levels and an increase in Zn concentrations. In the UK, Purvis et al. (2007) investigated 11 herbarium specimens of *Parmelia sulcata* collected between 1797 and 1967, comparing them with more recent collections (2000). Their findings indicated that older *Parmelia* specimens exhibited the highest levels of air pollution due to heavy metals and were also affected by dust contamination in the herbarium. In France, Agnan et al. (2013) analysed four herbarium specimens of *X. parietina* from the early 1900s and found them enriched with As, Pb, and Cd, likely due to coal combustion. In contrast, recently collected material reflected pollution (Cu, Pb, Sb, and Sn) associated with local factories and car traffic. In the Himalayan region, Bajpai et al. (2016) analysed herbarium specimens of *Heterodermia diademata* collected in 1966 from 11 localities and compared them with recent samples collected in 2014. The recent material was much richer in heavy metals and PAHs than the herbarium specimens, likely reflecting increased urbanisation of the study sites over the past five decades. Minganti et al. (2014) examined temporal trends of trace and rare earth elements in *Cetraria islandica* specimens from Italian herbaria. Their study analysed 24 specimens collected from remote and rural areas in Italy between 1981 and 2007, revealing a progressive decline in Pb concentrations due to the phase-out of leaded fuels, alongside a notable increase in Mn levels.

On the whole, the use of lichen collections offers both advantages and challenges. The main constraints can be summarised as follows:

1. The number of suitable specimens may be limited, restricting the ability to conduct comprehensive spatial and temporal comparisons, particularly when the selected species are rare or endangered.
2. Even when specimens are available, destructive analytical techniques inevitably influence the integrity of the material, both in terms of quantity and quality.
3. Older specimens may lack detailed information on collection locality or year, reducing the precision of comparisons.
4. Potential contamination from herbarium storage procedures must be carefully assessed.

Despite these limitations, it has been demonstrated that, with careful sampling and analysis, herbarium specimens can serve as invaluable resources for environmental science (Rudin et al. 2017). When other sources of data are unavailable, botanical collections provide critical insights into past environmental conditions and processes (e.g. Rudin et al. 2017; Shotbolt et al. 2007). In particular, lichens preserved in herbaria have proven to be effective tools for tracking historical patterns of trace element deposition, revealing changes driven by anthropogenic factors even in remote areas (Minganti et al. 2014).

## Conclusions

By analysing herbarium specimens of the model lichen *Lobaria pulmonaria*, we reconstructed air quality changes in remote forests of the Western Carpathians over six decades (1960–2022) and characterised historical and current background concentrations of selected heavy metals and metalloids. Several peaks of pollution were observed, especially up to 1989. Most of the elements showed a clear decrease with time, and the lowest values were recorded in recently collected specimens (2017–2022). The results reflected the progressive decrease of Pb content due to the ban of leaded fuels for vehicles, whose effects could be observed also in remote areas. Based on the available specimens, background concentrations have been estimated: the elemental content of *L. pulmonaria* between 1960 and 1989 in the Western Carpathians was at least four times higher than nowadays, while values measured until 1997 were almost three times higher. The study demonstrates that lichen herbarium specimens, even those collected from remote forested areas far from direct pollution sources, can serve as valuable indicators for reconstructing past environmental trends. The study highlights the potential of historical ecological archives, such as herbaria, as a resource for environmental monitoring.

## Supplementary Information

Below is the link to the electronic supplementary material.

Supplementary file 1 (PDF 5365 KB)

Supplementary file 2 (DOCX 15 KB)

## Data Availability

All data analysed during this study are included in the article.
