# Supplementary material for: Reconstructing Air Pollution Trends in Remote Forests of Central Europe Using Lichen Herbarium Specimens
Source: Arch Environ Contam Toxicol. 2025 Jun 21;89(1):34–45. doi: 10.1007/s00244-025-01134-9 (PMC12370840; doi:10.1007/s00244-025-01134-9)

Archives of Environmental Contamination and Toxicology

Reconstructing air pollution trends in remote forests of Central Europe using lichen herbarium specimens

Luca Paoli[a], Zuzana Fačkovcová[b,*], Anna Guttová[b]

a Department of Biology, University of Pisa, via L. Ghini 13, 56126 Pisa, Italy
b Plant Science and Biodiversity Centre, Slovak Academy of Sciences, Dúbravská cesta 9, 84523 Bratislava, Slovakia

*corresponding author (zuzana.fackovcova@savba.sk)

Supplementary information_S1: details on selected herbarium specimens of *Lobaria pulmonaria* from BRA and SAV collections (1960 – 1997) used for the analyses

Herbarium: BRA **Specimen:** 1. Strážov Mountains
code: 472 (BRACR6078) **date:** 9 July 1960 **altitude:** 1100 m **legit:** Ivan Pišút
locality: Strážov **substrate:** on *Fagus sylvatica*

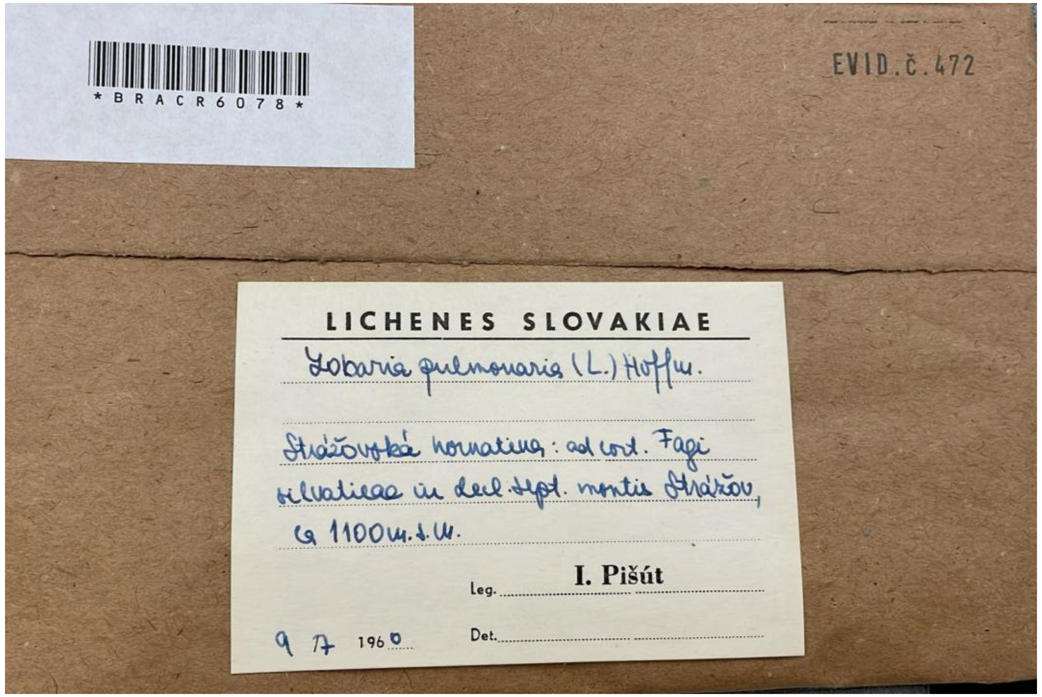

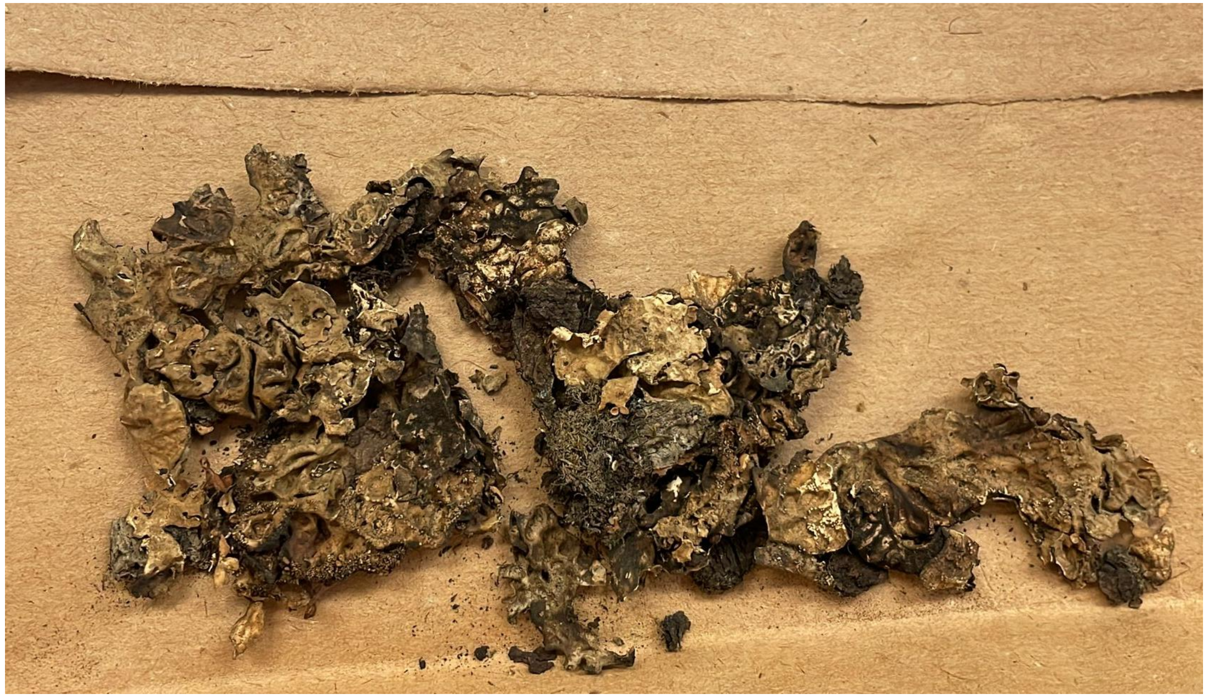

Herbarium: BRA **Specimen:** 2. Poloniny National Park, Bukovské vrchy Mts (Nízke Poloniny)
code: 10 **date:** 17 July 1962 **altitude:** 1190 m **legit:** Ivan Pišút
locality: Ďurkovec Mt. **substrate:** on *Acer pseudoplatanus*

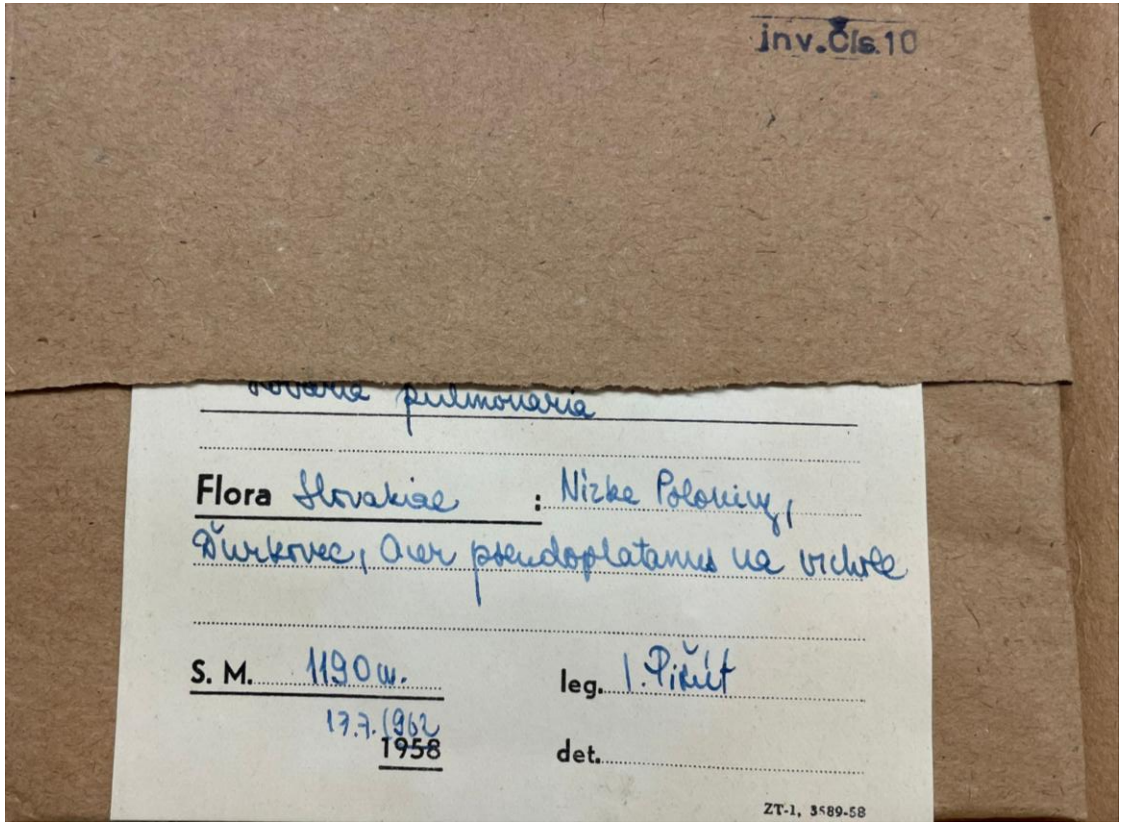

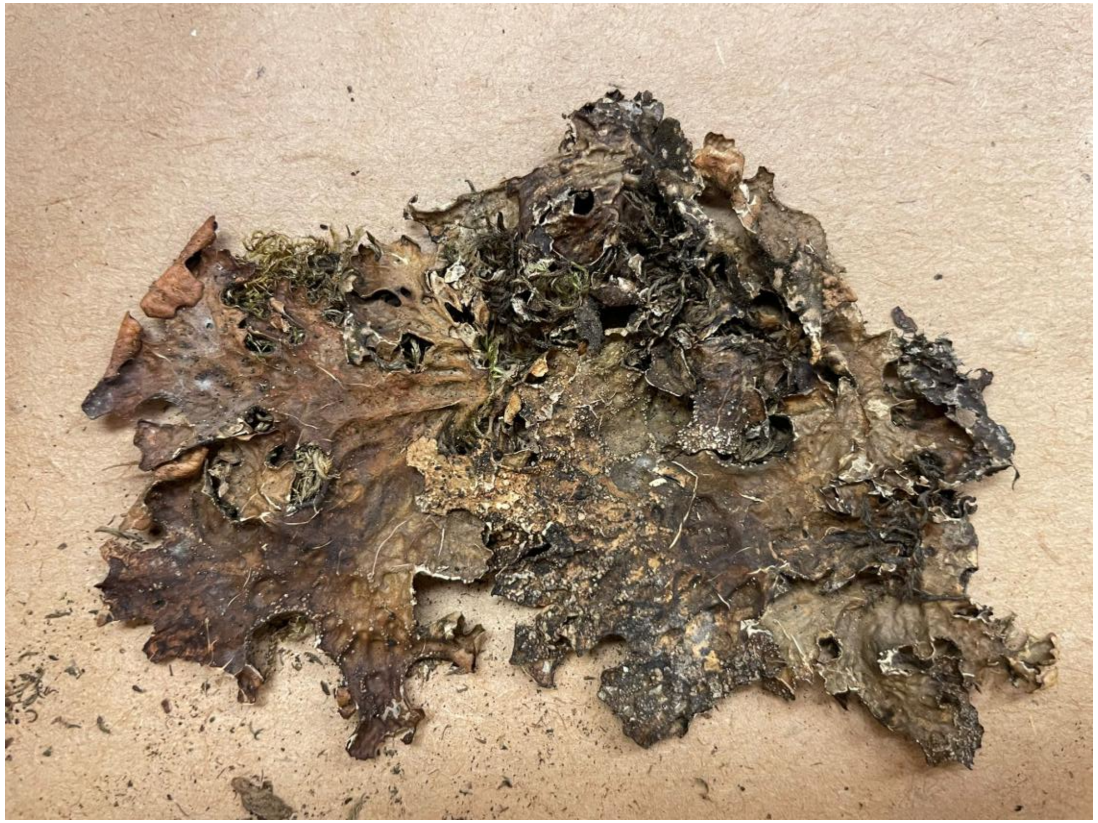

Herbarium: BRA **Specimen:** 3. Muránska planina National Park
code: 30 **date:** 24 September 1963 **altitude:** 930 m **legit:** Ivan Pišút
locality: Cigánka Mt., close to the ruins of the castle **substrate:** on *Ulmus glabra*

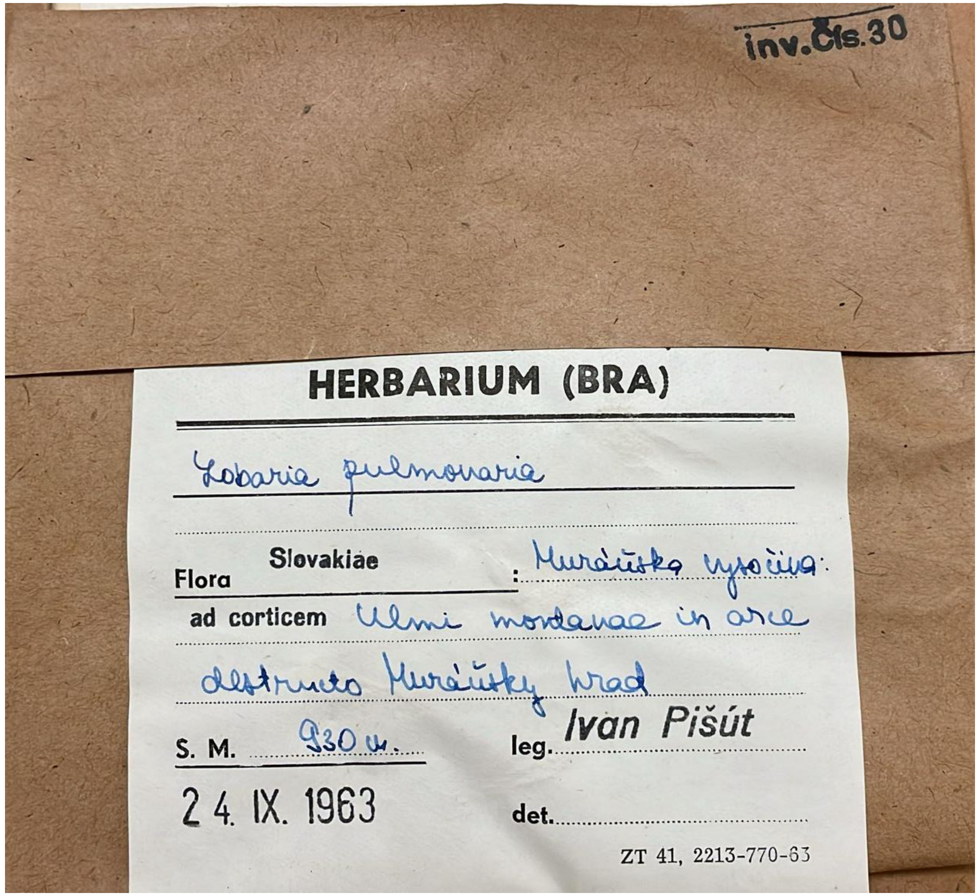

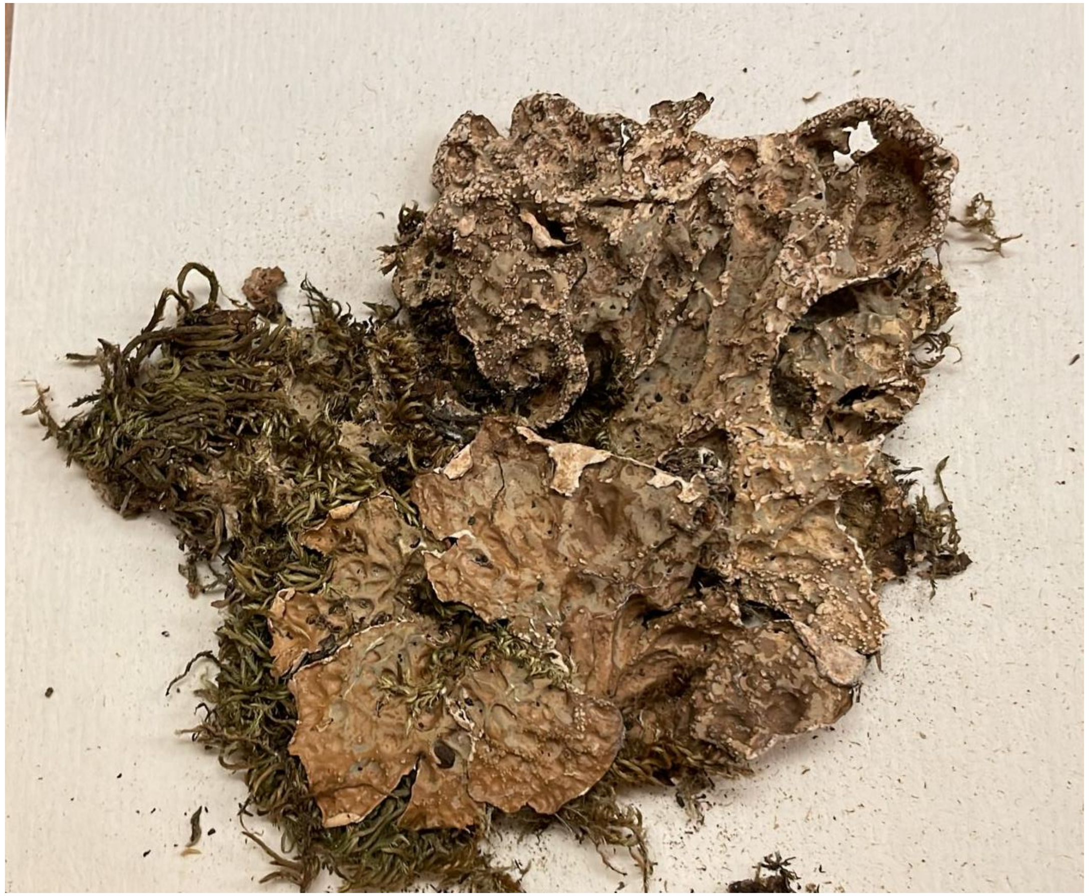

Herbarium: BRA **Specimen:** 4. Muránska planina National Park
code: 277 **date:** 1967 **altitude:** not reported (likely 1000 m) **legit:** Eva Jelínková
locality: Muráň plateau **substrate:** not available
notes: from the herbarium of Eva Lisická (Jelínková)

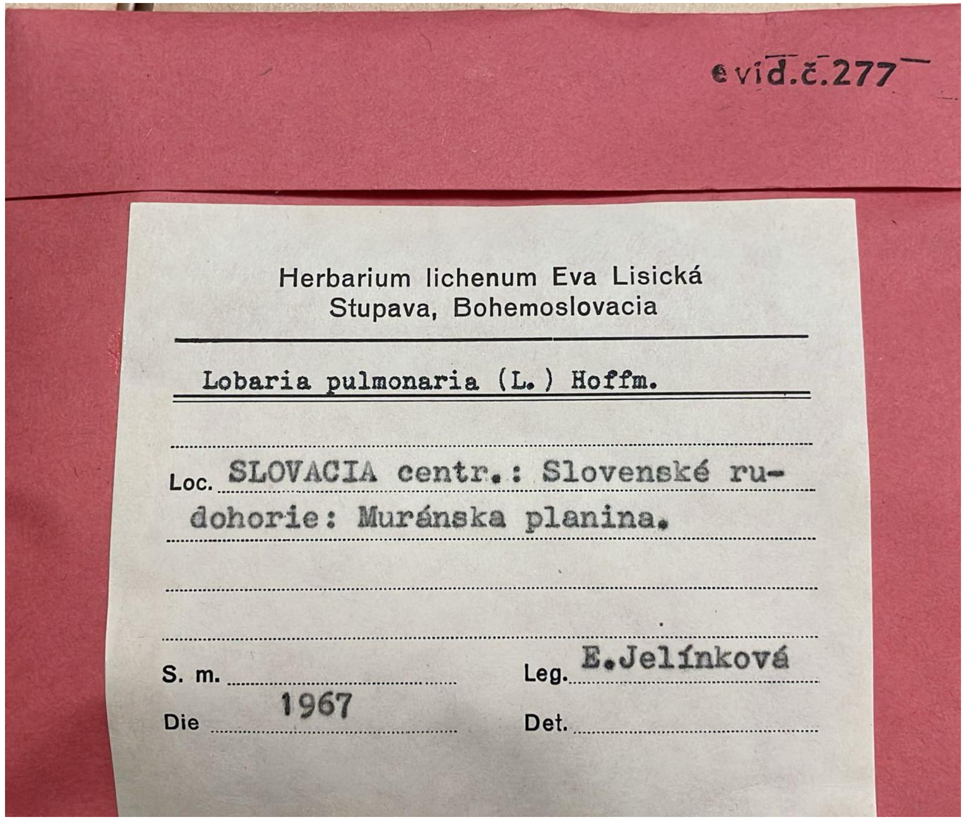

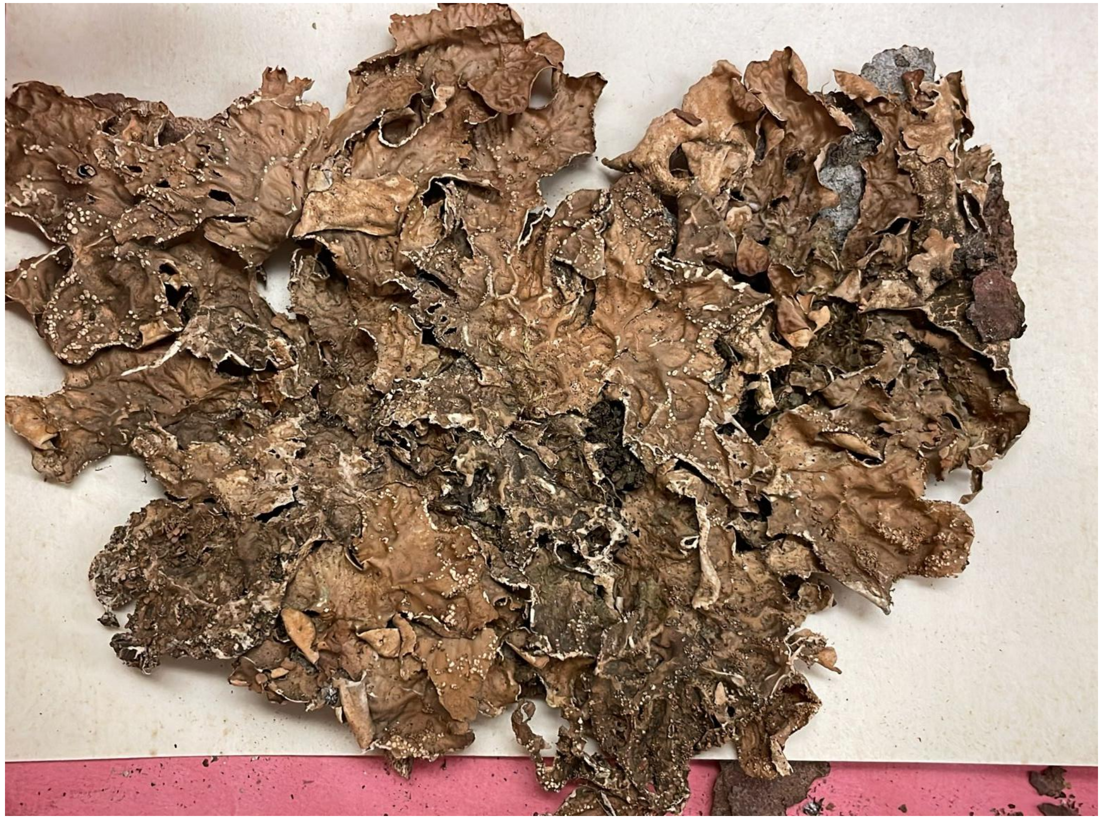

Herbarium: BRA **Specimen:** 5. Poloniny National Park
code: 345 **date:** 16 July 1969 **altitude:** 950 m **legit:** Eva Jelínková
locality: Ďurkovec Mt. **substrate:** on mosses on *Fagus sylvatica*
notes: from the herbarium of Eva Lisická (Jelínková)

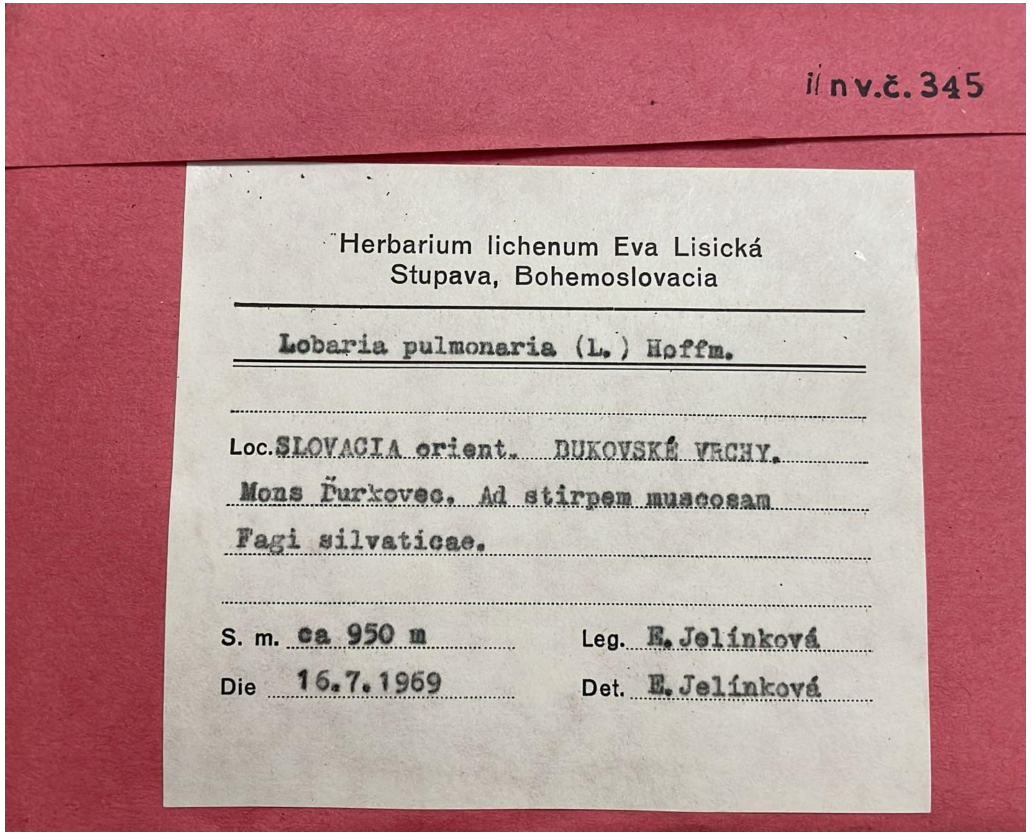

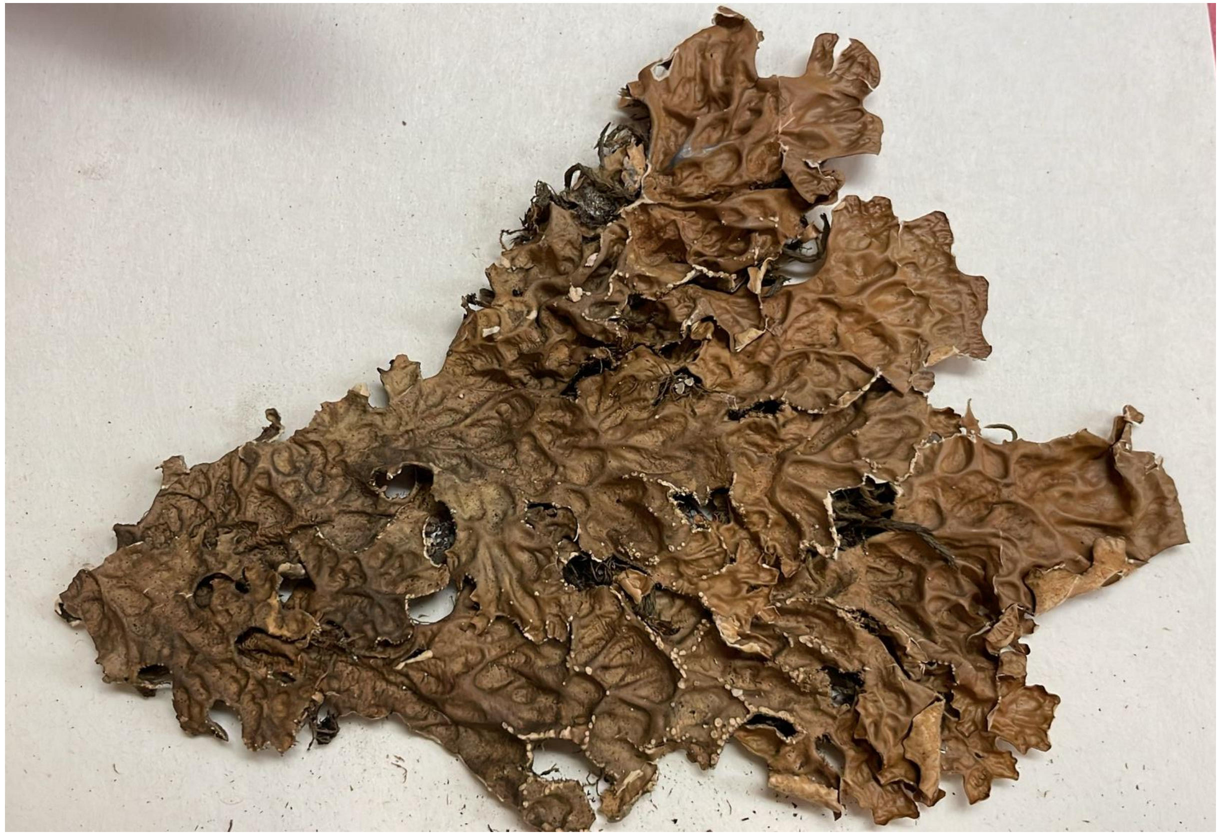

Herbarium: BRA **Specimen:** 6. Low Tatras National Park, Nízke Tatry Mts.
code: 114 **date:** 7 November 1972 **altitude:** 800 m **legit:** Ivan Pišút
locality: the valley Svarínska dolina **substrate:** on rock

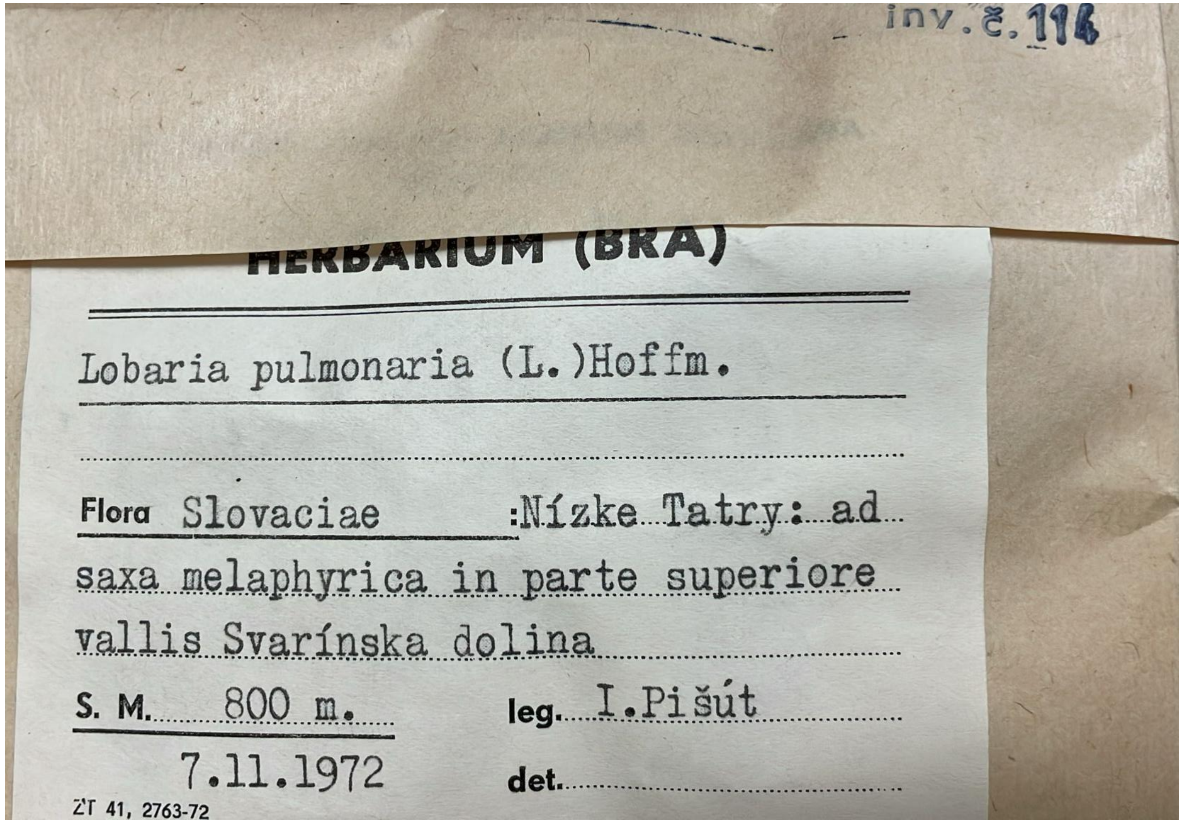

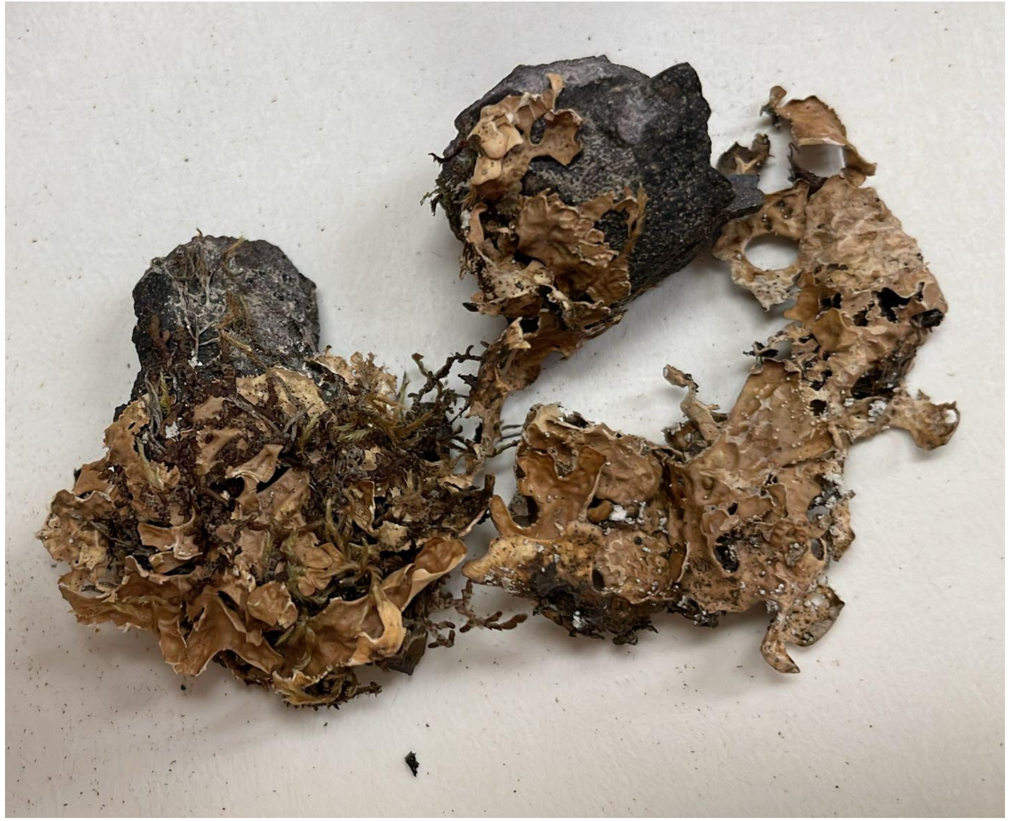

Herbarium: BRA **Specimen:** 7. Veľká Fatra National Park
code: 143 **date:** 5 June 1974 **altitude:** 850 m **legit:** Ivan Pišút
locality: the valley Dedošová dolina **substrate:** on *Acer pseudoplatanus*

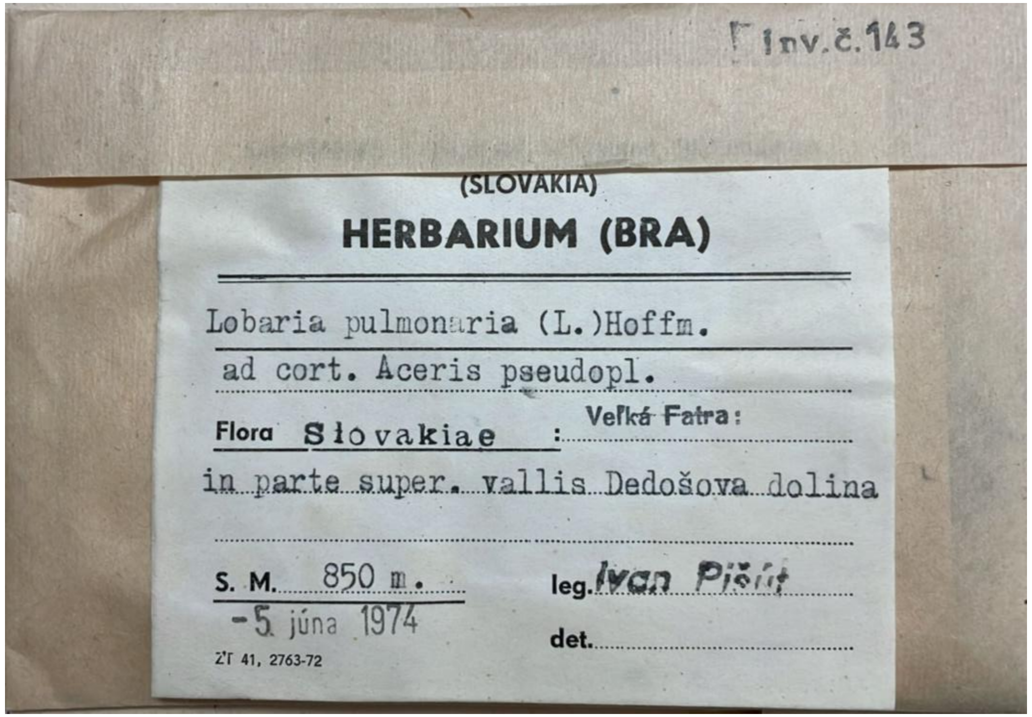
Inv. č. 143

(SLOVAKIA)

HERBARIUM (BRA)

Lobaria pulmonaria (L.)Hoffm.
ad cort. Aceris pseudopl.
Flora Slovakiae : Veľká Fatra :
in parte super. vallis Dedošova dolina

S. M. 850 m. leg. Ivan Pišút
-5 júna 1974 det.
ZT 41, 2763-72

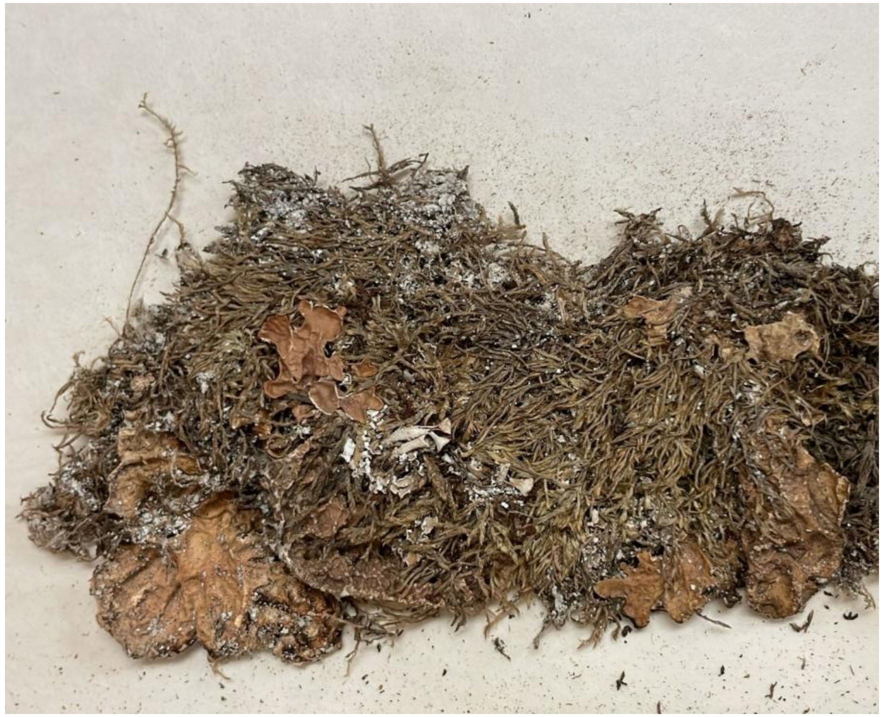

Herbarium: BRA **Specimen:** 8. Poloniny National Park, Bukovské vrchy Mts.
code: 225 **date:** 7 July 1980 **altitude:** 800 m **legit:** Ivan Pišút
locality: Nová Sedlica, the valley Stužická rieka **substrate:** on *Fagus sylvatica*

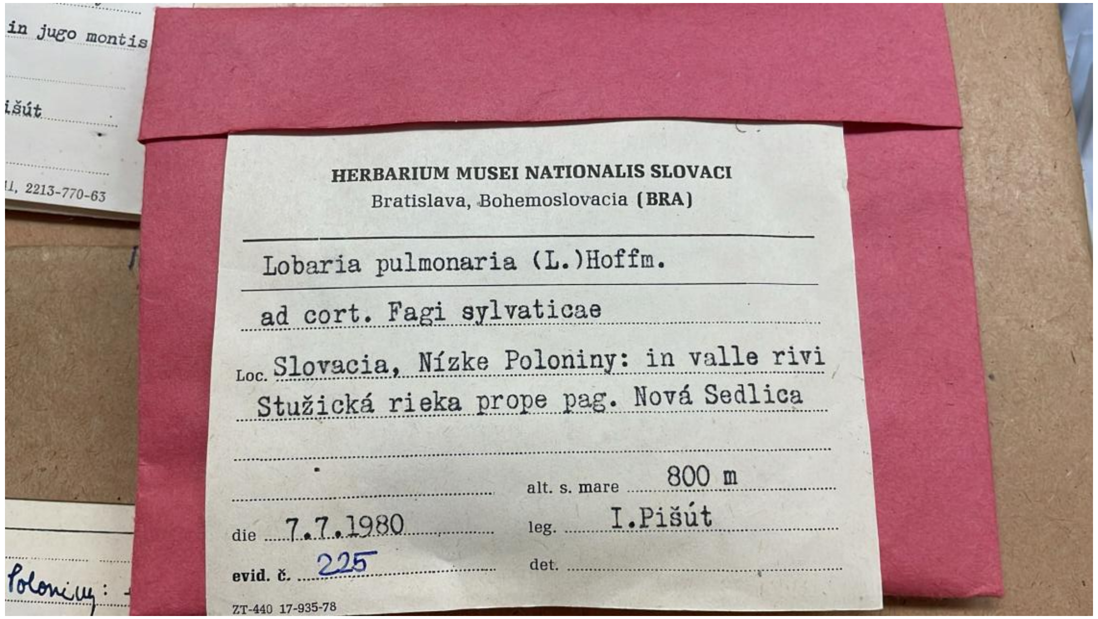

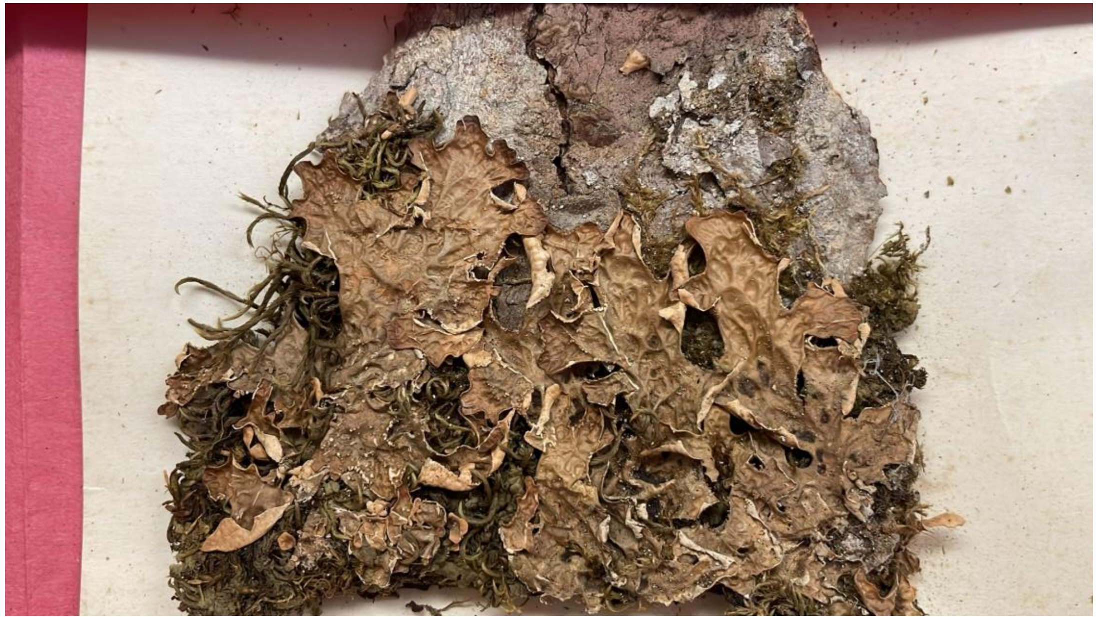

Herbarium: BRA **Specimen:** 9. Muránska planina National Park
code: 322 **date:** 20 June 1988 **altitude:** 900 m **legit:** M. Svetlíková
locality: Cigánka Mt. **substrate:** on bark (tree not specified)

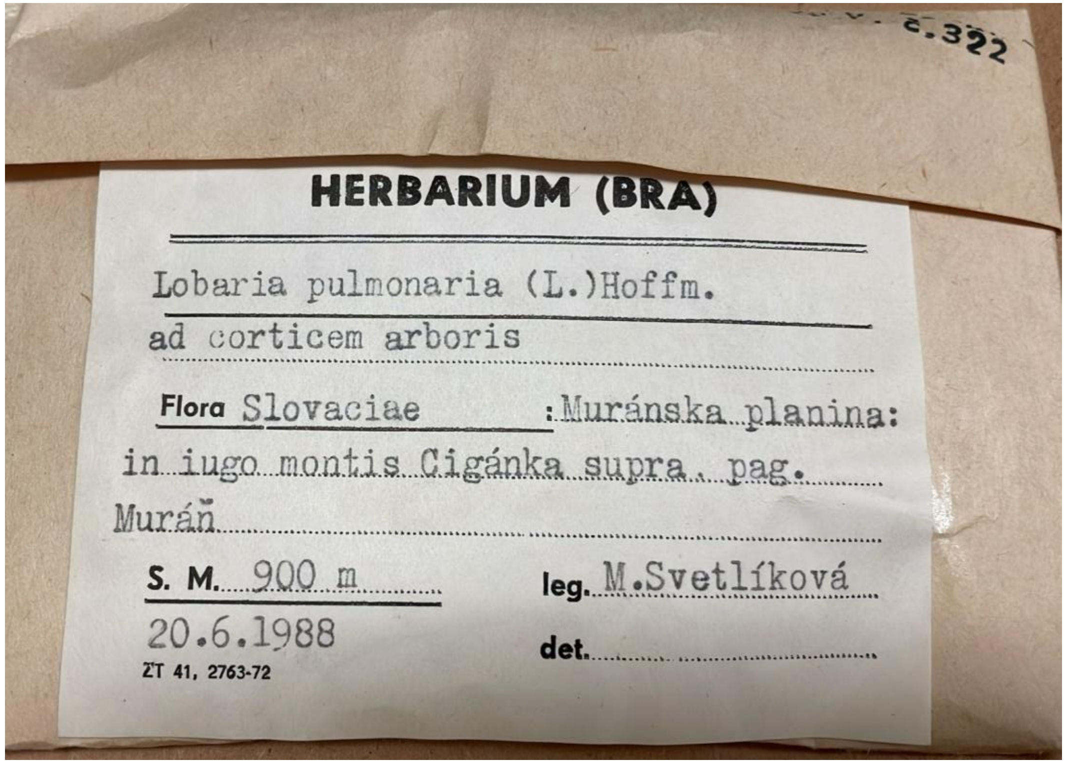
č.322

HERBARIUM (BRA)

Lobaria pulmonaria (L.)Hoffm.
ad corticem arboris

Flora Slovaciae :Muránska planina:
in iugo montis Cigánka supra. pag.
Muráň

S. M. 900 m leg. M.Svetlíková
20.6.1988 det.
ZT 41, 2763-72

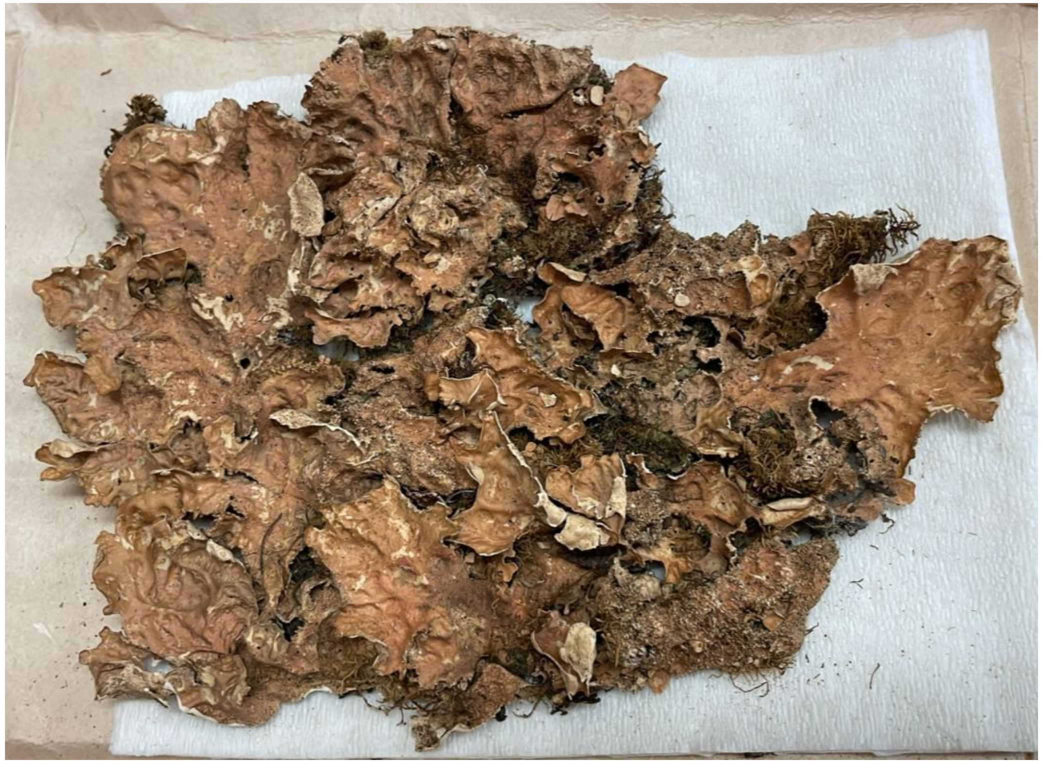

Herbarium: SAV **Specimen:** 10. Poloniny National Park, Bukovské vrchy Mts.
code: - **date:** 22 August 1989 **altitude:** 1070 m **legit:** Ivan Pišút
locality: Čierťaž Mt. **substrate:** on *Fagus sylvatica*

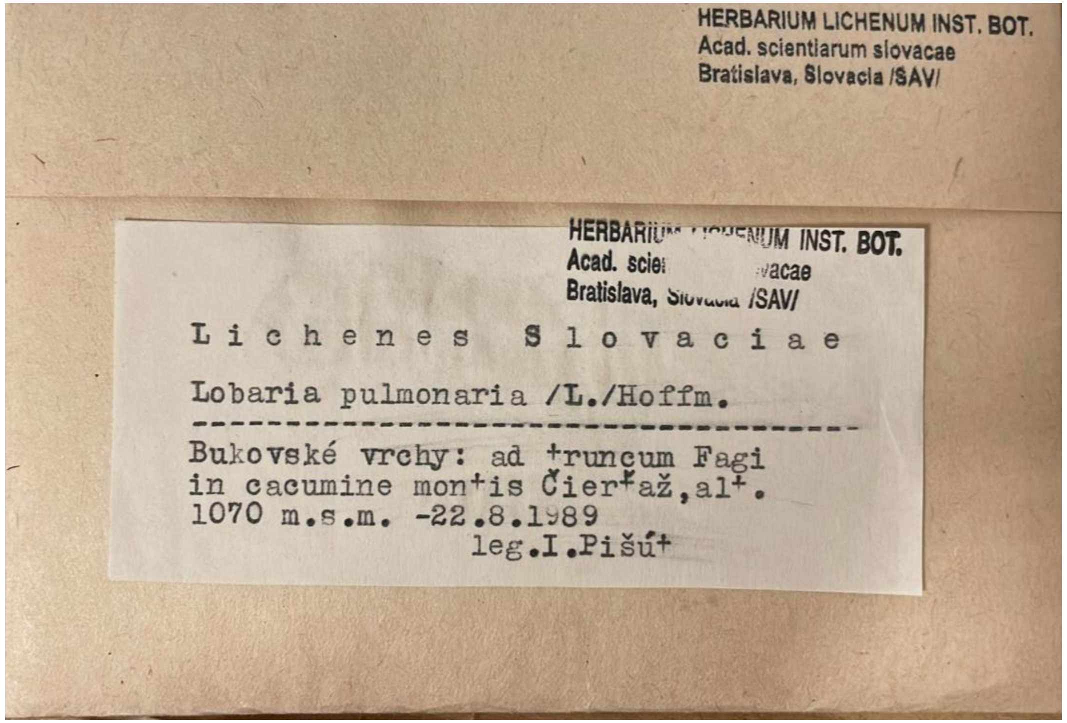

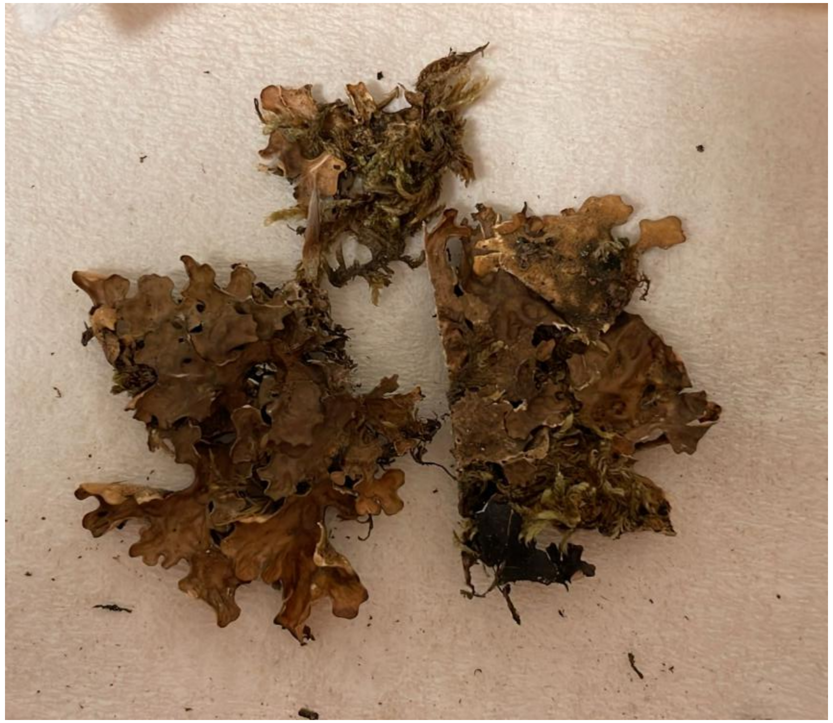

Herbarium: SAV **Specimen:** 11. Veľká Fatra National Park
code: - **date:** 17 October 1995 **altitude:** 650 m **legit:** Ivan Pišút
locality: Blatnica, the valley Gaderská dolina **substrate:** on *Salix caprea*

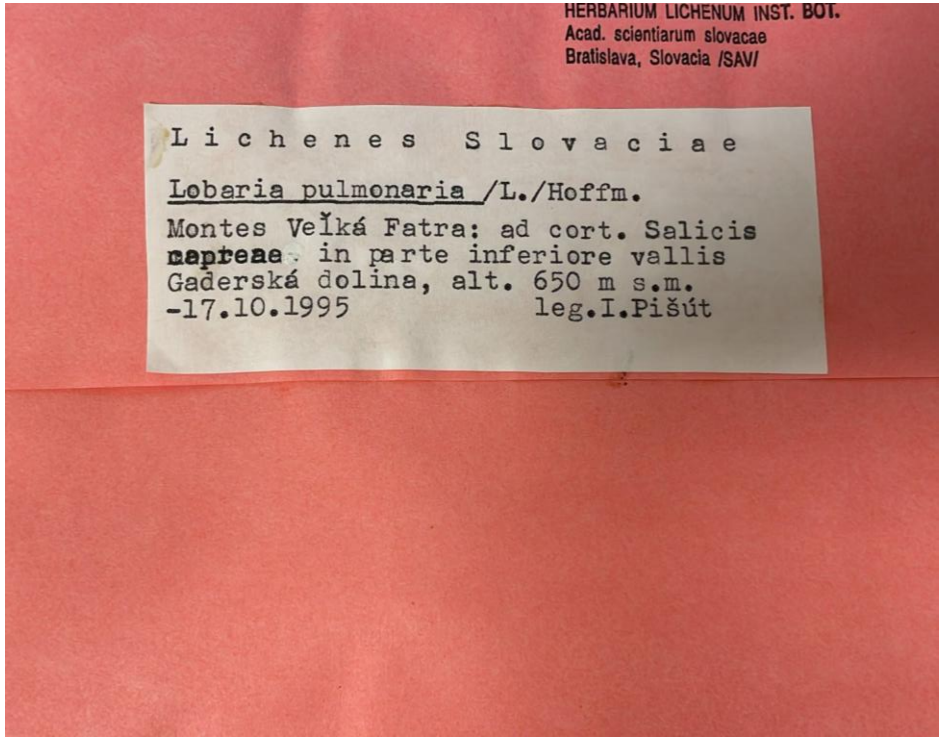

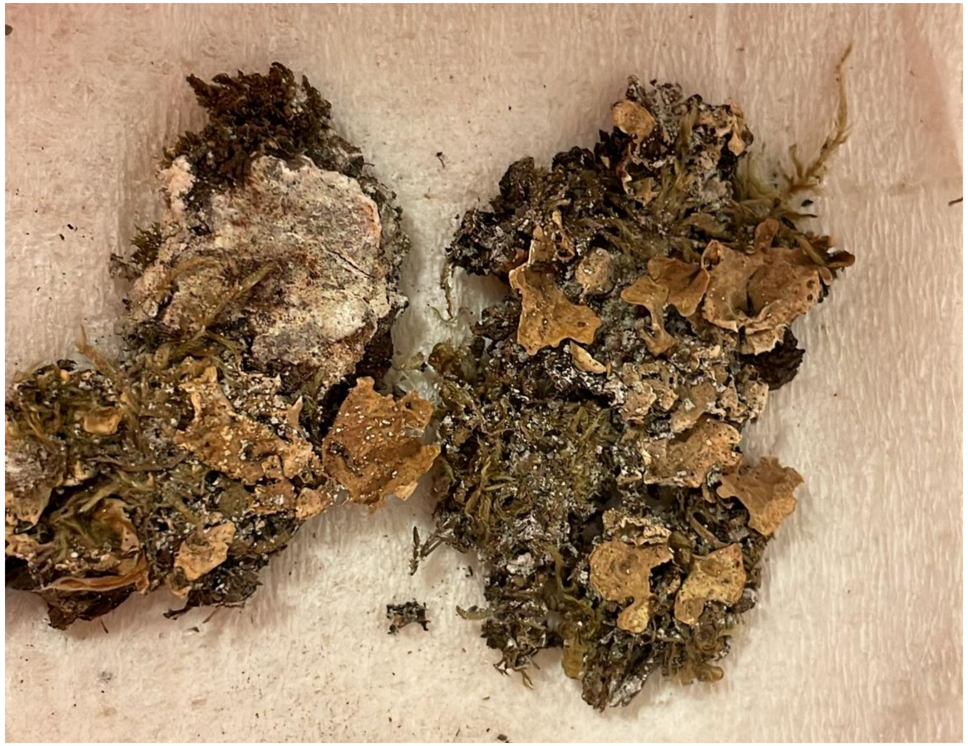

Herbarium: SAV **Specimen:** 12. Muránska planina National Park
code: - **date:** 28 September 1995 **altitude:** 800 m **legit:** Ivan Pišút
locality: Závadka nad Hronom, the valley Za Nihovo **substrate:** on *Ulmus glabra*

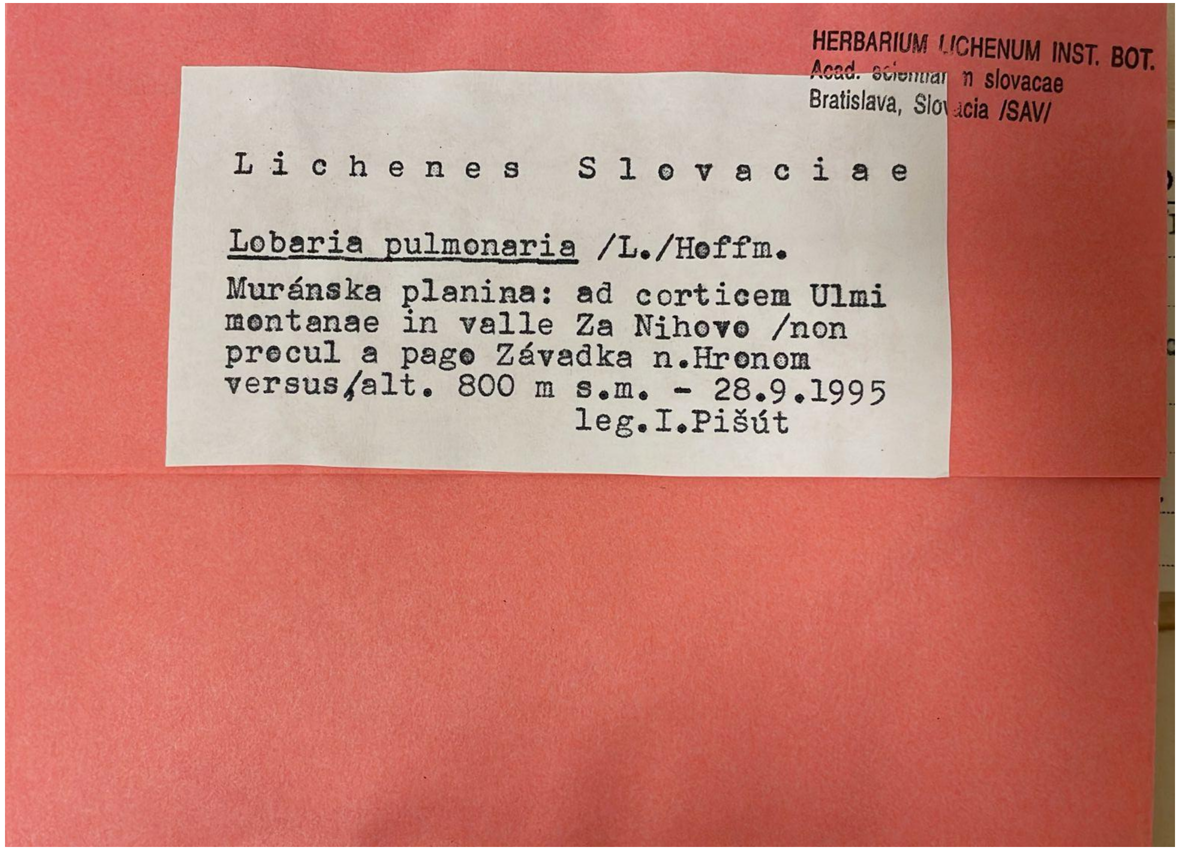

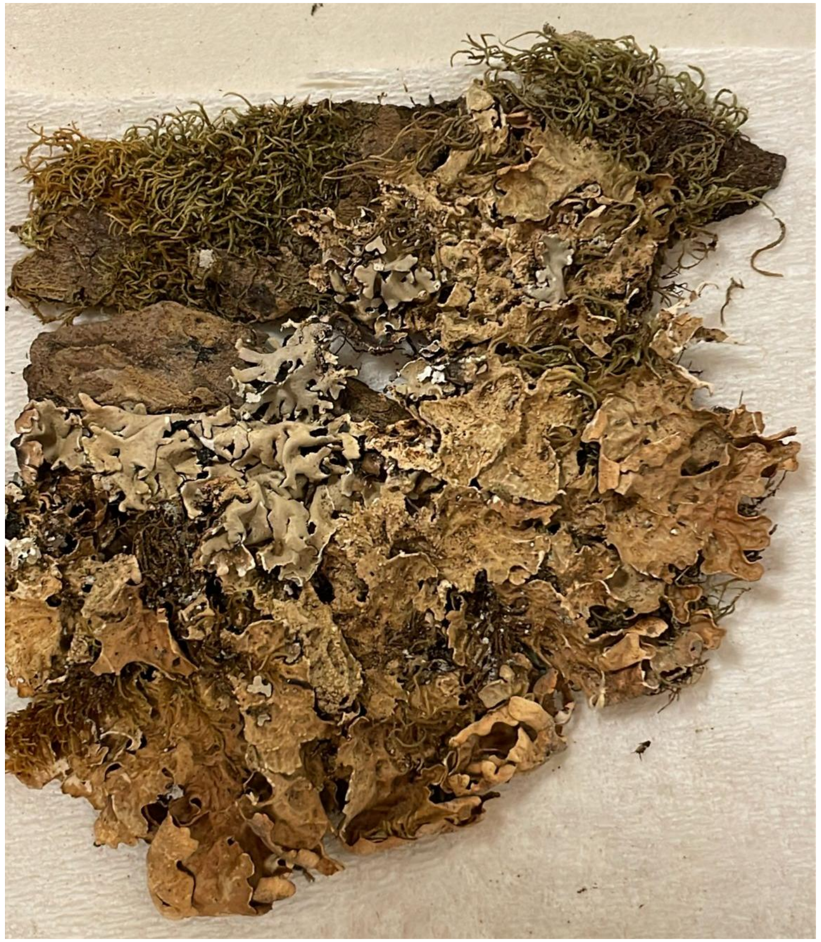

Herbarium: SAV **Specimen:** 13. Muránska planina National Park
code: - **date:** May 1997 **altitude:** 920 m **legit:** A. Guttová, Z. Palice
locality: Cigánka Mt., close to the ruins of the castle **substrate:** on *Fraxinus excelsior*

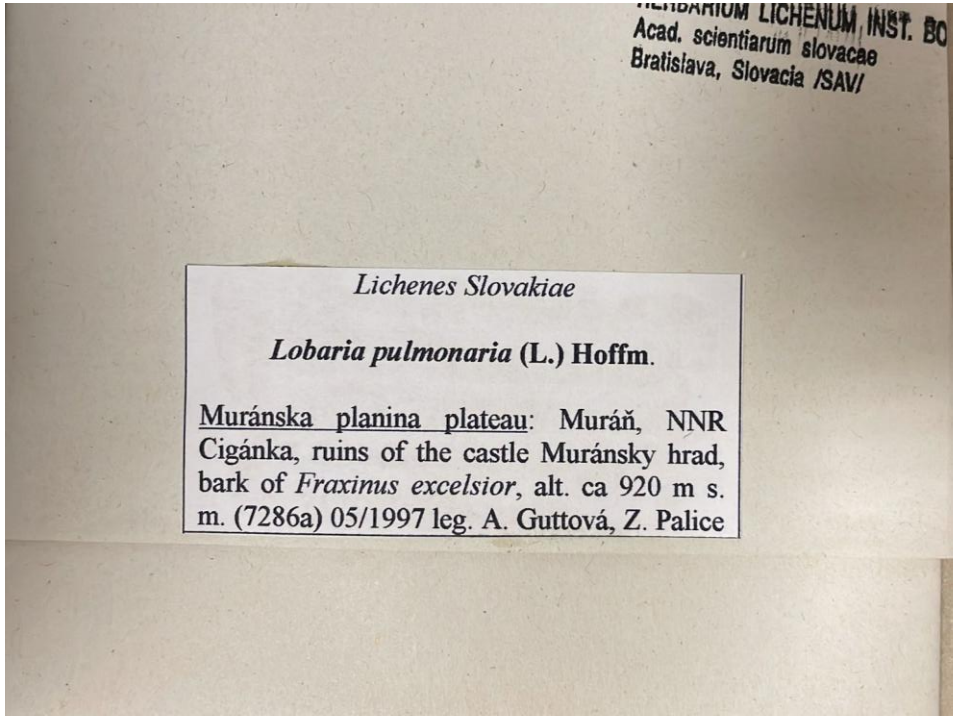

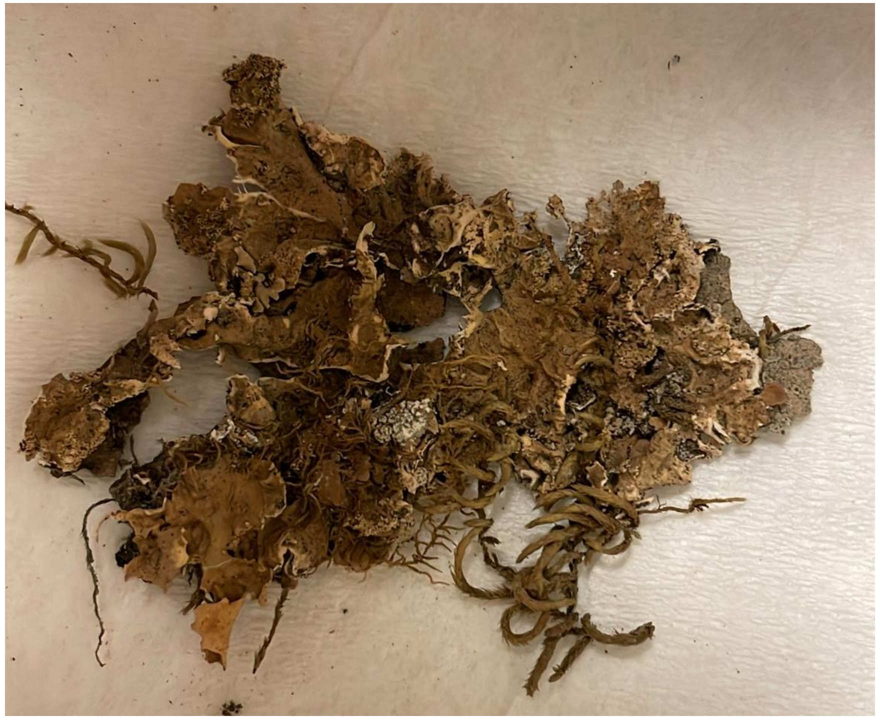

Herbarium: BRA **Specimens:** Malé Karpaty Mts. (Little Carpathians)
code: 57 **date:** not available **altitude:** not available **legit:** Karol Mergl
localities: Pressburg (today Bratislava), Blumenau (today Lamač) **substrate:** not available
Notes: specimens excluded from the dataset due to Hg contamination.
In the middle, detail of fruiting bodies (apothecia) in a fertile thallus.

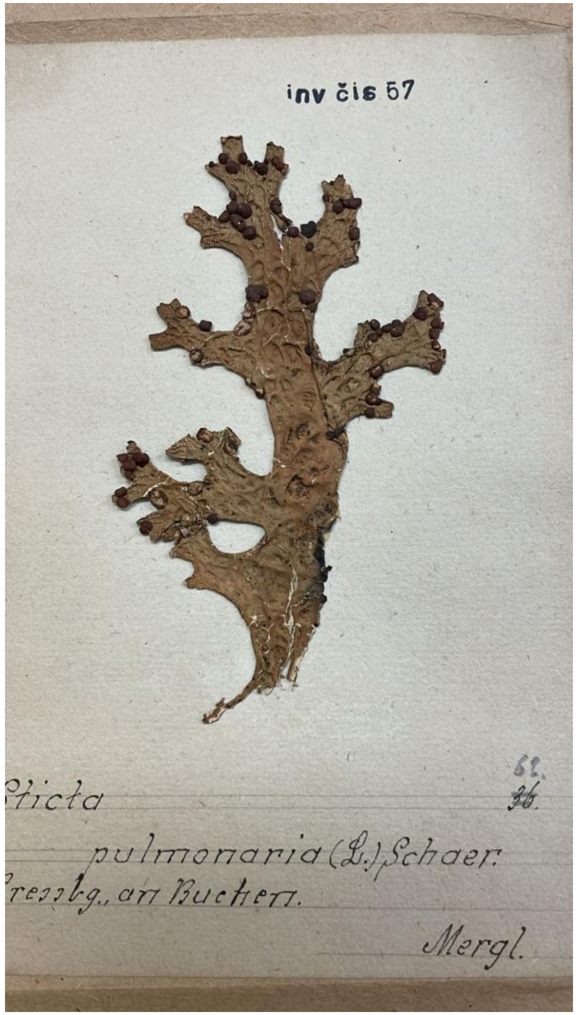

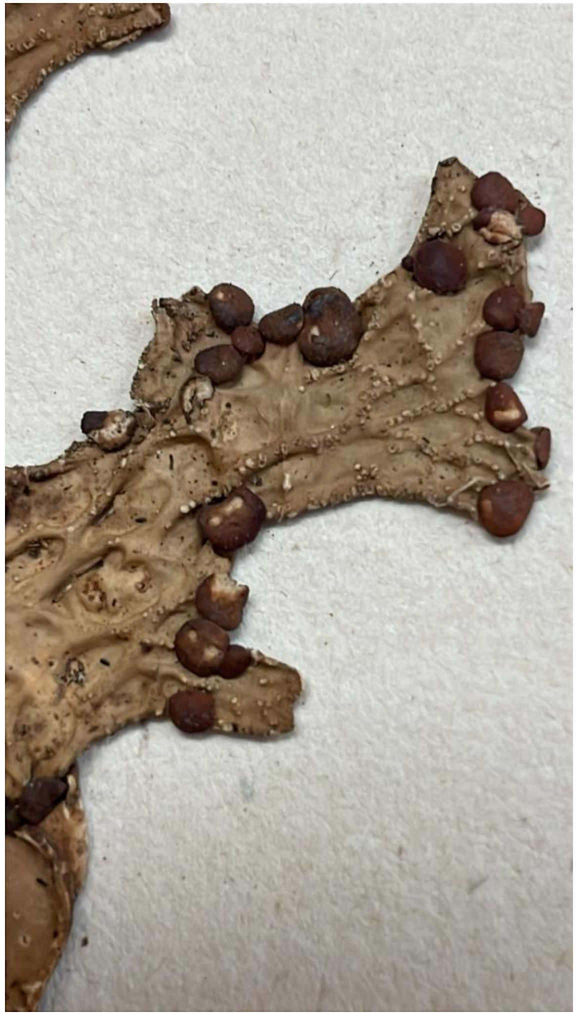

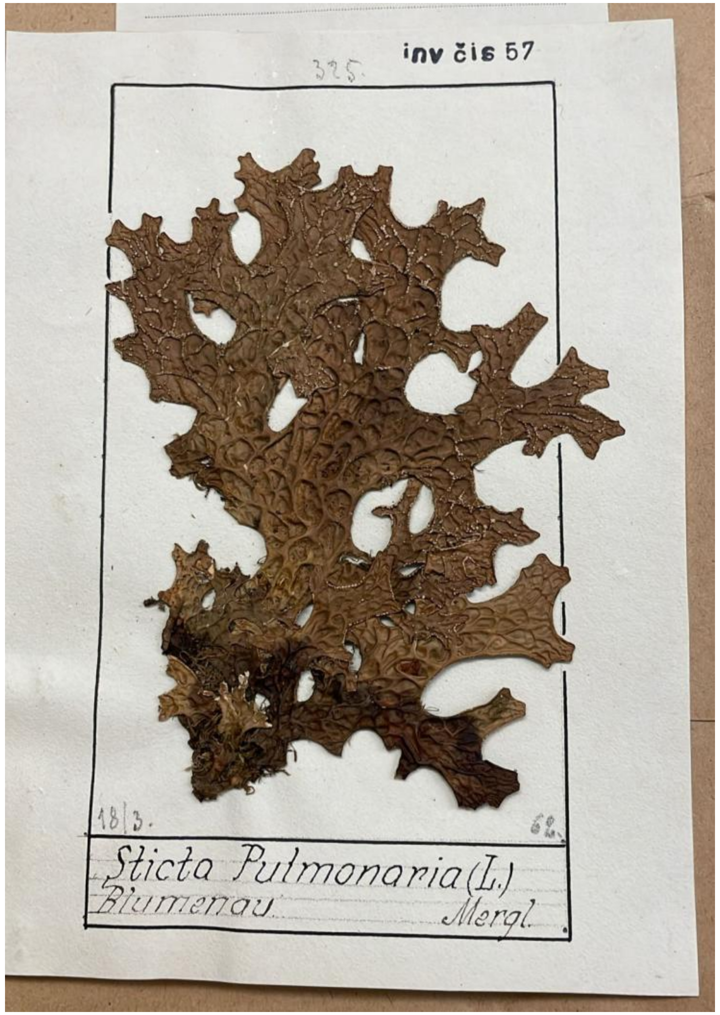

Supplement: Supplementary file 1 — (PDF 5365 KB) [file 244_2025_1134_MOESM1_ESM.pdf]
