# Supplementary material for: Reconstructing Air Pollution Trends in Remote Forests of Central Europe Using Lichen Herbarium Specimens
Source: Arch Environ Contam Toxicol. 2025 Jun 21;89(1):34–45. doi: 10.1007/s00244-025-01134-9 (PMC12370840; doi:10.1007/s00244-025-01134-9)
Supplement: Supplementary file 2 — (DOCX 15 KB) [file 244_2025_1134_MOESM2_ESM.docx]

| Element | Limit of Quantification | IAEA-336  average (min-max) | Replicate 1 | Replicate 2 | Replicate 3 |
| --- | --- | --- | --- | --- | --- |
| Al | 0.16 | 680 (570 – 690) | 589 | 596 | 598 |
| As | 0.02 | 0.6 (0.55 – 0.71) | 0.69 | 0.63 | 0.64 |
| Cd | 0.01 | 0.12 (0.100 – 0.134) | 0.12 | 0.11 | 0.10 |
| Cr | 0.05 | 1.06 (0.89 – 1.23) | 1.08 | 0.97 | 1.20 |
| Cu | 0.01 | 3.6 (3.1 – 4.1) | 3.9 | 4.0 | 4.1 |
| Fe | 1.35 | 430 (380 – 480) | 414 | 450 | 449 |
| Mn | 0.05 | 63 (56 – 70) | 58 | 57 | 60 |
| Pb | 0.004 | 4.9 (4.3 – 5.5) | 4.4 | 4.3 | 4.4 |
| Sb | 0.005 | 0.073 (0.063 – 0.083) | 0.071 | 0.069 | 0.072 |
| Zn | 0.27 | 30.4 (27.0 – 33.8) | 31.8 | 28.8 | 31.1 |
| Element | Limit of Quantification | GBW-07604  average ± SD | Replicate 1 | Replicate 2 | Replicate 3 |
| Hg | 0.0003 | 0.026 ± 0.003 | 0.030 | 0.025 | 0.025 |
| Ni | 0.024 | 1.90 ± 0.30 | 2.19 | 2.04 | 1.93 |
| S | 60 | 3500 ± 300 | 3570 | 3593 | 3627 |

***Archives of Environmental Contamination and Toxicology***

**Reconstructing air pollution trends in remote forests of Central Europe using lichen herbarium specimens**

Luca Paoli^a^, Zuzana Fačkovcová^b,*^, Anna Guttová^b^

a Department of Biology, University of Pisa, via L. Ghini 13, 56126 Pisa, Italy

b Plant Science and Biodiversity Centre, Slovak Academy of Sciences, Dúbravská cesta 9, 84523 Bratislava, Slovakia

*corresponding author ([zuzana.fackovcova@savba.sk](mailto:zuzana.fackovcova@savba.sk))

Supplementary information_S2: Concentrations (µg/g dw) of potentially toxic elements analysed by ICP-MS. Values were determined from three independent replicates of Standard Reference Materials: IAEA-336 (lichen; Al, As, Cd, Cr, Cu, Fe, Mn, Pb, Sb, Zn) and GBW-07604 (poplar leaves; Hg, Ni, S).
